# Silica sulfuric acid coated on SnFe_2_O_4_ MNPs: synthesis, characterization and catalytic applications in the synthesis of polyhydroquinolines†

**DOI:** 10.1039/d2ra01202b

**Published:** 2022-05-12

**Authors:** Soheila Esmaili, Ardeshir Khazaei, Arash Ghorbani-Choghamarani, Masoud Mohammadi

**Affiliations:** Department of Organic Chemistry, Faculty of Chemistry, Bu-Ali Sina University Hamedan 6517838683 Iran ardeshir_khazaei@yahoo.com a.ghorbani@basu.ac.ir arashghch58@yahoo.com; Department of Chemistry, Faculty of Science, Ilam University P.O. Box 69315516 Ilam Iran

## Abstract

An efficient and heterogeneous novel magnetic solid sulfuric acid, immobilized on silica functionalized SnFe_2_O_4_, was successfully synthesized, characterized, and employed as a novel recoverable nanocatalyst for the synthesis of biologically active polyhydroquinoline derivatives. The SnFe_2_O_4_@SiO_2_–SO_3_H was easily synthesized and confirmed using various spectroscopic techniques, including FT-IR, XRD, EDX, Map, TGA, SEM and TEM analyses. The catalytic behavior of the resulting catalyst system was investigated in the Hantzsch synthesis of polyhydroquinoline derivatives. The desired products were obtained with high conversions and excellent reusability.

## Abstract 2

An efficient and heterogeneous novel magnetic solid sulfuric acid, immobilized on silica functionalized SnFe_2_O_4_, was successfully synthesized, characterized, and employed as a novel recoverable nanocatalyst for the synthesis of biologically active polyhydroquinoline derivatives.

## Graphical abstract

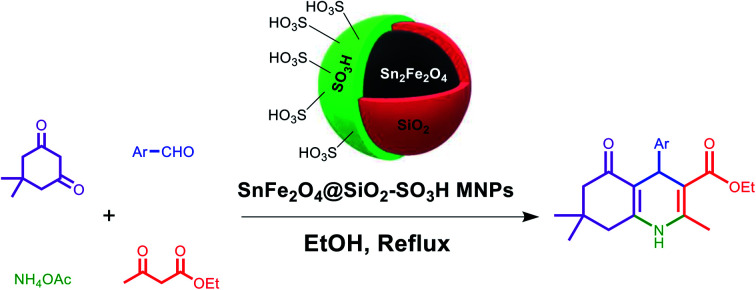

## 1. Introduction

Asymmetric Hantzsch synthesis of polyhydroquinolines includes the catalytic Knoevenagel condensation–Michael addition–cyclization sequence in which 1 eq. of dimedone is heated with aldehyde derivatives in the presence of ethyl acetoacetate and an ammonia source.^1–3^ In this sense, a multi-step sequence ensues, a water molecule is lost and the target six-membered nitrogen-containing scaffold is formed.^4,5^ It possesses a chiral center at the phenyl-substituted carbon.^6–9^ We have previously reviewed the biological and pharmacological activities and the available synthetic methods for the synthesis of polyhydroquinoline derivatives.^1^

Catalysis science is considered as the main center of most key organic reactions such as the named reactions,^10,11^ most of the key organic functional group transformations require a catalyst in the reaction media to the selective conversion of the reagents and synthons to the target products with high performance.^9,12–14^ In this sense, the utilization of heterogeneous nanomaterials as catalysts has attracted worldwide attention due to their unique role in the conversion of these manufacturing procedures to ecofriendly, greener, economical and viable methods.^15–20^

During recent decades, acid catalysts have played the main role in the organic functional group transformations especially in multicomponent reactions, despite the widespread use of organic and inorganic acid catalysts, the leaching of hazardous acids into the desired product is one of the negative aspects of employing heterogeneous acid-based catalysts in the sustainable catalysis.^1^ To overcome this problem, the coupling of homogeneous acid catalysts with heterogeneous catalytic materials, as the catalyst, seems to be a suitable solution.^12,21^ In this case, the catalytic support role is to properly distribute the acid cites to operate the special properties of these moieties.^12,21^

Magnetically separable nanomaterials which can be considered as one of the most important classes of materials with unique physicochemical properties have attracted the attention of a wide variety of researchers.^22–24^ Regarding the catalytic support materials, these spinel ferrite compounds have great potential in industry and technology as green heterogeneous catalysts in various organic functional group transformations and as catalytic supports.^25–30^ Based on our interest in developing heterogeneous catalysts with the use of nanomaterials, we have recently reported the synthesis of novel heterogeneous catalytic supports that were functionalized by organic and inorganic ligands and complexes.^1,12,20,31,32^ In the continuation of our studies, we wish to report the spinel normal SnFe_2_O_4_ as a versatile nanomagnetic catalytic support, for the synthesis of a novel supported silica sulfuric acid catalyst, which is the first report on the utilization of SnFe_2_O_4_ MNPs as the catalytic support.

Considering the interesting benefits of heterogeneous catalysts with the use of novel and green materials, herein, we reported the synthesis of an efficient and heterogeneous novel silica sulfuric acid coated on SnFe_2_O_4_ MNPs and its application in the asymmetric Hantzsch synthesis of polyhydroquinolines in high yields under mild conditions.

## 2. Experimental

### 2.1. Preparation of sulfuric acid supported on the surface of SnFe_2_O_4_ MNPs

**Scheme 1:**
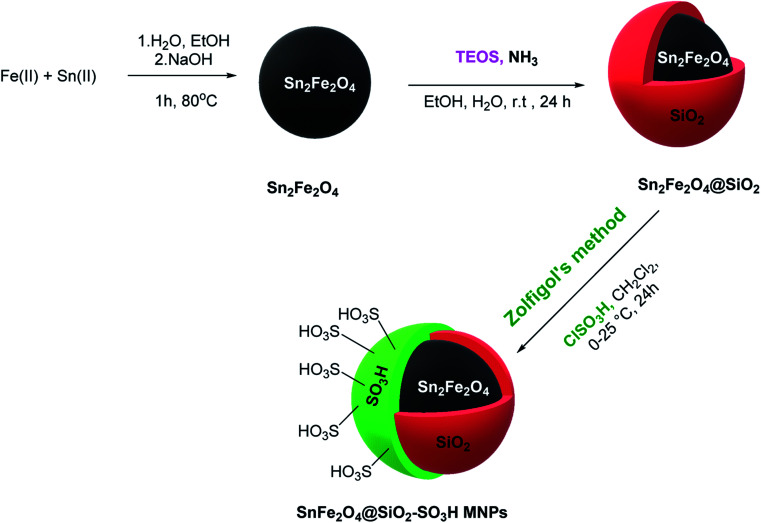
Synthesis of SnFe_2_O_4_@SiO_2_–SO_3_H MNPs.

The SnFe_2_O_4_ magnetic nanoparticles were prepared by the coprecipitation technique as was previously reported.^33^ Afterward, its surface was coated with SiO_2_ shell according to the previously reported method by our group.^13^ Afterward, the sulfuric acid catalytic cites graphed on its surface according to Zolfigol's method: 1.5 mL of chlorosulfuric acid was added dropwise to a dispersed mixture of SnFe_2_O_4_ MNPS (1 g) in dry CH_2_Cl_2_ (20 mL) and the reaction was cooled in an ice bath, then, the mixture was stirred for 4 h at room temperature. Finally, the obtained SnFe_2_O_4_@silica sulfuric acid MNPs were separated using an external magnet, washed by dry CH_2_Cl_2,_ and dried at 80 °C in an oven for 12 h (Scheme 1).

### 2.2. General procedure for the catalytic synthesis of polyhydroquinolines

A mixture of aromatic aldehydes (1.0 mmol), ethyl acetoacetate (1 mmol), dimedone (1 mmol), NH_4_OAc (1.2 mmol), and SnFe_2_O_4_@SiO_2_–SO_3_H (12 mg) was stirred in 3 mL ethanol under reflux conditions for the required time. The progress of the reaction was monitored by TLC. After completion of the reaction, the reaction mixture was diluted with hot ethanol to dissolve the organic products. Afterward, the catalyst was collected by magnetic decantation. Finally, the pure polyhydroquinoline products were obtained through recrystallization in ethanol and washed with diethyl ether.

## 3. Results and discussions

### 3.1. Catalyst characterization

The as-prepared SnFe_2_O_4_@SiO_2_–SO_3_H and its parent core-shells were then fully characterized using different physio-chemical techniques, including; FT-IR, XRD, TGA, VSM, EDX, X-ray-mapping, and SEM analysis.

**Fig. 1:**
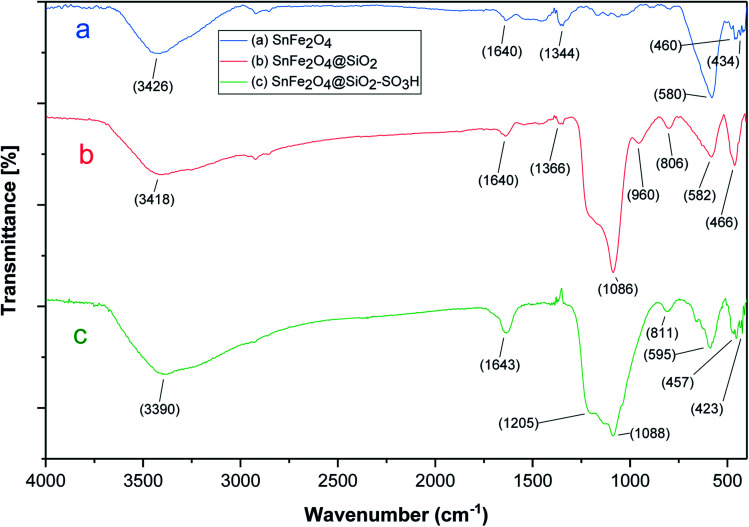
The FT-IR spectra of SnFe_2_O_4_, SnFe_2_O_4_@SiO_2,_ and SnFe_2_O_4_@SiO_2_–SO_3_H MNPs.

FT-IR analysis (Fig. 1) shows the FT-IR spectra of SnFe_2_O_4_, SnFe_2_O_4_@SiO_2,_ and SnFe_2_O_4_@SiO_2_–SO_3_H MNPs. All FT-IR spectra in Fig. 1 are completely consistent with the previous analyses of SnFe_2_O_4_ MNPs,^33^ indicating bands around 3426 cm^−1^ and 1640 cm^−1^ (hydroxyls, interlayer water molecules stretching vibrations). Moreover, peaks at around 580 and 460 cm^−1^ are formed by the stretching vibrations of the Sn–O, and Fe–O bonds in spinel ferrite structures, respectively. In SnFe_2_O_4_@SiO_2_ spectra, the characteristic bonds at 1086 cm^−1^ (Si–O) and 806 cm^−1^ (Si–O–Si) confirm the successful coating of silica shells on the surface of the MNPs and the formation of the corresponding core–shell composition. In the SnFe_2_O_4_@SiO_2_–SO_3_H spectra, finally, the boarding and overlapping of the peaks around the 850–1300 cm^−1^ and 2700–3700 cm^−1^ bands in FT-IR spectra of SnFe_2_O_4_@sulfuric acid (Fig. 1c) confirm the successful functionalization of SnFe_2_O_4_@SiO_2_ core–shell with the SO_3_H functional groups.^34^

**Fig. 2:**
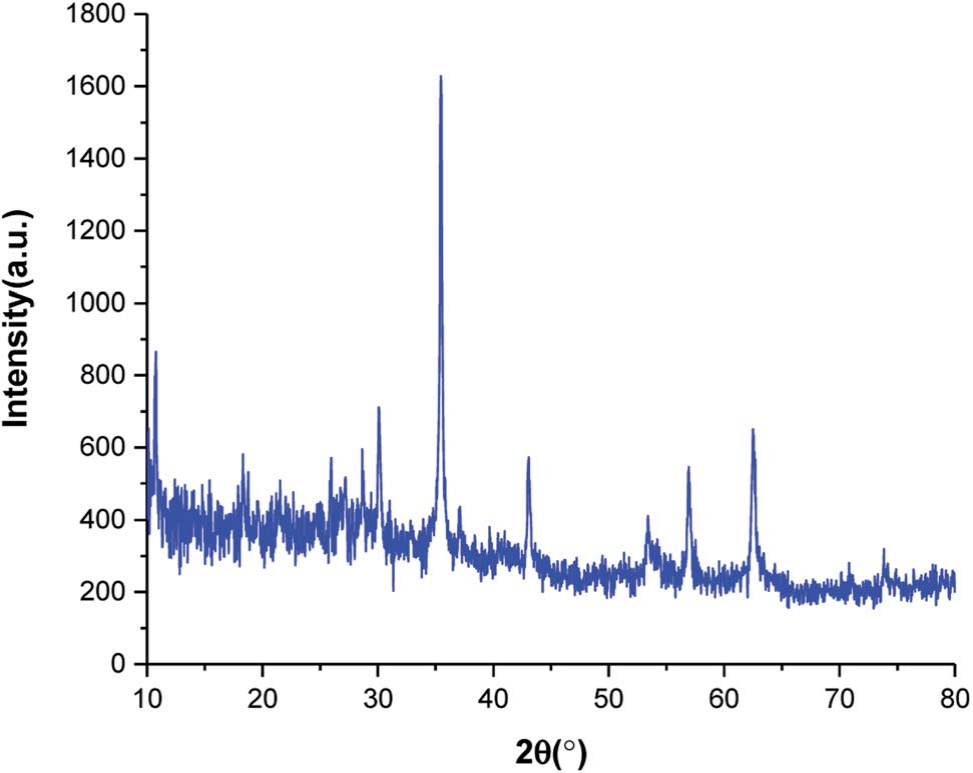
XRD pattern of SnFe_2_O_4_@SiO_2_–SO_3_H MNPs.

The crystalline phase of SnFe_2_O_4_@SiO_2_–SO_3_H MNPs was examined *via* the XRD analysis. As shown in Fig. 2, the SnFe_2_O_4_@SiO_2_–SO_3_H MNPs afforded seven sharp and strong peaks at 2*θ* = 30.1, 35.45, 36.89, 43.12, 53.27, 56.88 and 62.45 indexed to the (220), (311), (222), (400), (422), (511) and (440) planes, respectively showing good agreement with XRD pattern of previous reports on SnFe_2_O_4_ MNPs.^33^ These results confirm that the tubular structure of SnFe_2_O_4_ is not destroyed during the functionalization and stabilization of the silica sulfuric acid shell, and the noisy background coming from the amorphous dried SO_3_H shells. Finally, the average crystalline size of SnFe_2_O_4_@SiO_2_–SO_3_H MNPs calculated from the Scherrer equation is 17.43 nm.^35^

**Fig. 3:**
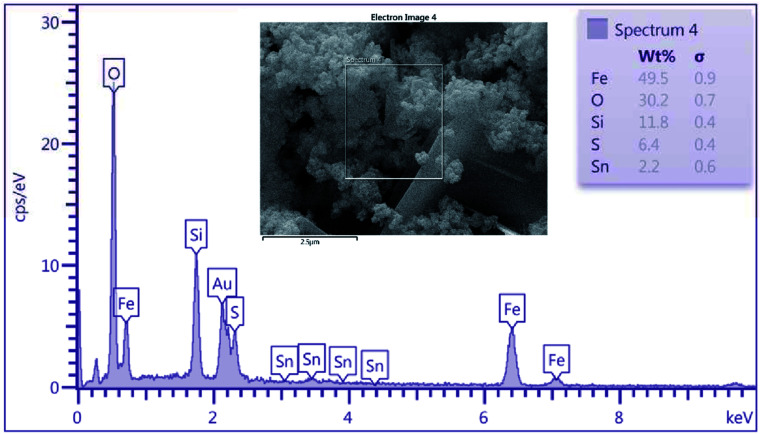
EDS analysis of SnFe_2_O_4_@SiO_2_–SO_3_H MNPs.

As shown in Fig. 3, energy dispersive X-ray (EDX) analysis was applied to determine the chemical composition of nanoporous SnFe_2_O_4_@SiO_2_–SO_3_H MNPs. The results indicate the presence of Sn, Fe, and O species in the obtained spinel ferrite catalyst. Besides, the successful grafting of SiO_2_ shell over the SnFe_2_O_4_ catalytic support was confirmed by the presence of Si species. The existence of sulfur in the SnFe_2_O_4_@SiO_2_–SO_3_H MNPs nanocatalyst was considered by the EDX spectrum, but we did not observe any amount of Cl, indicating that it was on the catalyst surface where the covalent adsorption of SO_3_H groups has successfully occurred. Besides, the Cl was removed as HCl gas from the reaction vessel, immediately. These observations support the high purity of the prepared catalyst. According to this EDX spectrum, it could be inferred that the target catalyst has been successfully synthesized. Moreover, the exact amount of sulfuric acid loading on SnFe_2_O_4_@SiO_2_–SO_3_H was 6.4 wt%.

**Fig. 4:**
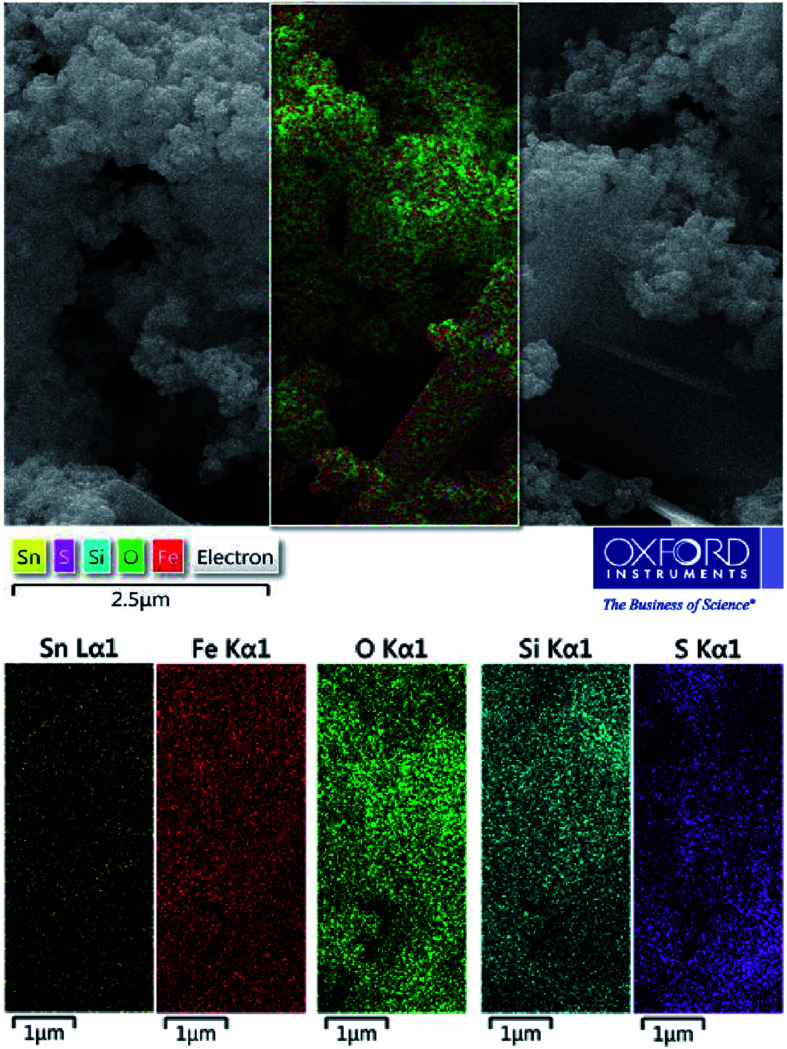
X-ray-mapping analysis of SnFe_2_O_4_@SiO_2_–SO_3_H MNPs.

To complete the elemental characterizations, the elemental mapping analysis was conducted for the investigation of elements distribution on the SnFe_2_O_4_@SiO_2_–SO_3_H MNPs (Fig. 4). According to this compositional map, obtained data confirmed the existence of Sn, Fe, O, Si, and S elements in the as-prepared nanomaterial with a suitable and homogeneously dispersity throughout the matrix surface. In this sense, the uniform distribution of active sulfuric acid sites on the SnFe_2_O_4_@SiO_2_ surface has a significant impact on the catalytic performance because of the good availability of the sulfonated Brønsted acid catalytic sites. Hence, the obtained result from the elemental mapping technique confirmed the obtained result from EDX analysis.

**Fig. 5:**
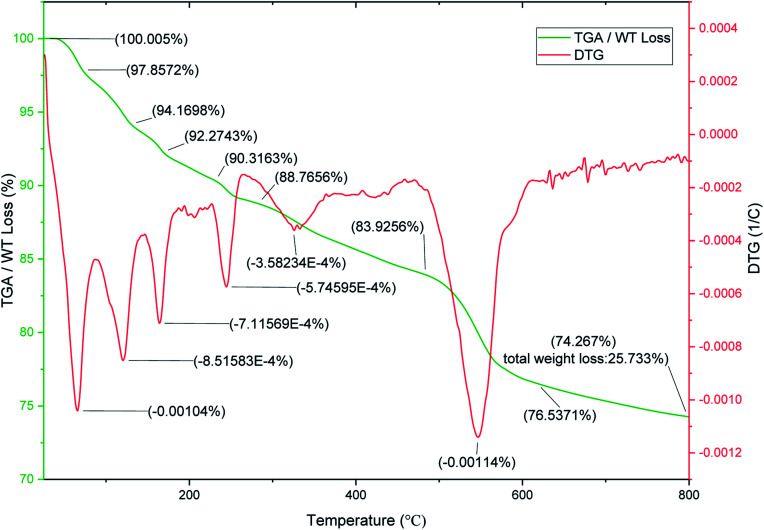
TGA/DTG curves of SnFe_2_O_4_@SiO_2_–SO_3_H MNPs.

To evaluate the thermal stability of SnFe_2_O_4_@SiO_2_–SO_3_H MNPs, the TGA and DTG analysis over the temperature range of 25–800 °C was investigated (Fig. 5). The TGA curve indicates the three-weight loss for SnFe_2_O_4_@SiO_2_–SO_3_H MNPs. The first weight loss of about 9.68% occurred below 200 °C which can be attributed to the release of the physically adsorbed moisture, water, and organic solvents from the sample.^36^ The next weight loss (6.39%) in the region of 200–480 °C can be associated with the removal of hydroxyl groups as water molecules on the surface of attached silanol groups during the pyrolysis process. The final weight loss (8.61%) at the region of 480–700 °C is attributed to the SO_3_H groups. The results confirm the successful chemical adsorption of silica sulfuric acid *via* chemical bonding on the SnFe_2_O_4_ nanomagnetic support. The DTG analysis has multistep patterns and confirms the core–shell structure of the magnetic silica gel coated SO_3_H catalytic system with various layers.

**Fig. 6:**
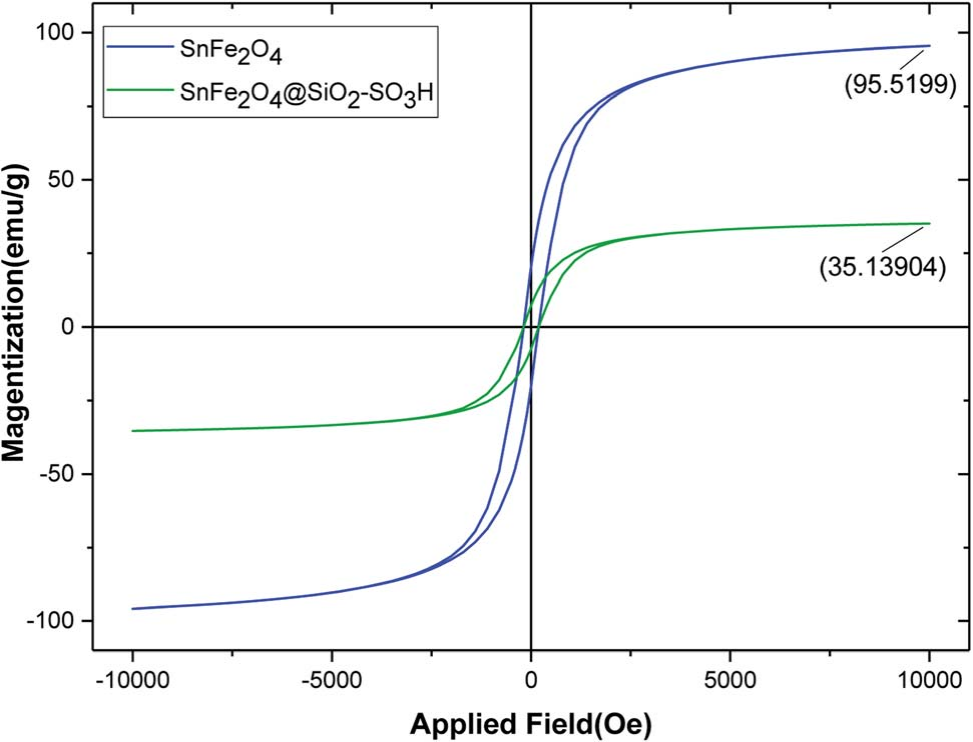
VSM curves of SnFe_2_O_4_ (blue) and SnFe_2_O_4_@SiO_2_–SO_3_H MNPs (green).

Magnetic properties of uncoated magnetic spinel ferrite–tin oxide (SnFe_2_O_4_) and SnFe_2_O_4_@SiO_2_–SO_3_H MNPs were studied by VSM analysis in the external magnetic range of −10 000 to +10 000 Oe at room temperature (Fig. 6). As can be seen from Fig. 5, the magnetic hysteresis curves of SnFe_2_O_4_ and SnFe_2_O_4_@SiO_2_–SO_3_H MNPs show their super magnetic behavior, and the magnetizations are 95.51, 45.80, and 30.90 emu g^−1^, respectively. The reduction of magnetic strength from 56.80 to 35.13 is due to the successful addition of the SiO_2_ shell and sulfuric acid compounds. Nevertheless, SnFe_2_O_4_@SiO_2_–SO_3_H has suitable and excellent magnetization values that can be quickly separated from the solution through an external magnetic field.

**Fig. 7:**
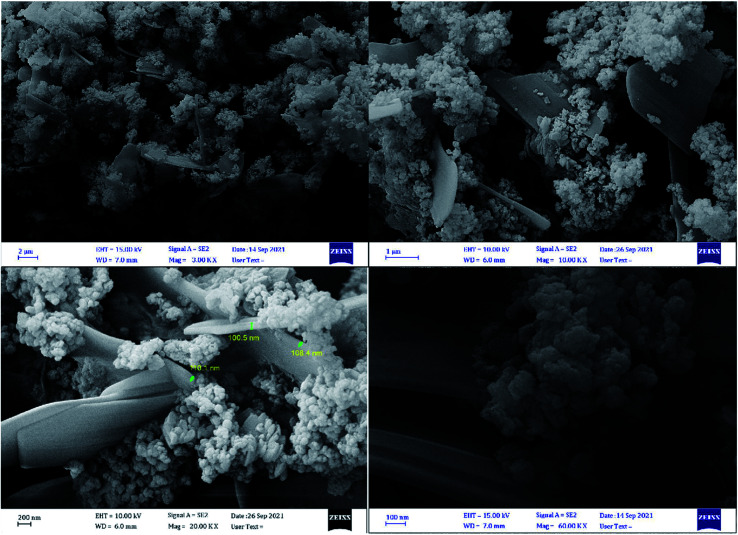
SEM images of SnFe_2_O_4_@SiO_2_–SO_3_H MNPs.

The structural features, particle size, and morphologies of the SnFe_2_O_4_@SiO_2_–SO_3_H MNPs were identified with FE-SEM images (Fig. 7). The FE-SEM images illustrate that the as-prepared nanocomposite is in an almost regular spherical shape and has an average size of 15–20 nm. The rougher structures of SnFe_2_O_4_@SiO_2_–SO_3_H rather than SnFe_2_O_4_ (ref. 37) can be attributed to the successful surface coating of organic compounds. Moreover, it is obvious from Fig. 7 that the average size of SnFe_2_O_4_ is significantly changed after immobilization of SiO_2_ shell and coating with SO_3_H, which the successful synthesis of SnFe_2_O_4_@SiO_2_–SO_3_H.

**Fig. 8:**
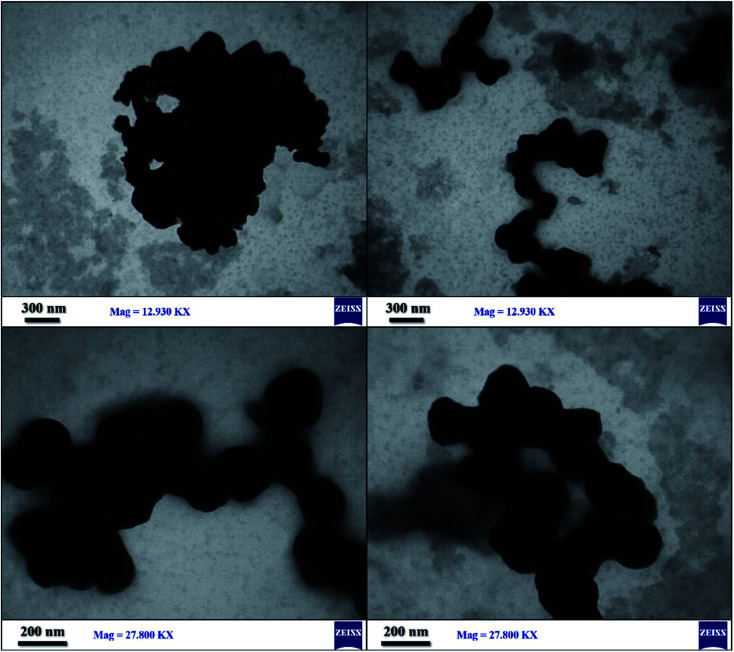
TEM images of SnFe_2_O_4_@SiO_2_–SO_3_H MNPs.

The size distribution of SnFe_2_O_4_@SiO_2_–SO_3_H MNPs was analyzed by transmission electron microscopy technique (Fig. 8). High-quality images from the synthesized crystalline SnFe_2_O_4_@SiO_2_–SO_3_H were obtained and the corresponding images attest that the silica with the bright area was successfully coated on SnFe_2_O_4_with the dark area. Moreover, a clear gap, between the shell and the support, confirms that the support is a solid sphere. Also, due to the magnetic attraction between the particles, a stacking texture, and slight aggregation can be observed. Meanwhile, the loading of sulfuric acid did not affect the morphology of the support and, as can be seen, the synthesized catalyst exhibited some specific characteristics of the crystalline structure. Also, TEM images like the SEM images verified the spherical shape of SnFe_2_O_4_@SiO_2_–SO_3_H.

### 3.2. Catalytic studies

The catalytic efficiency of heterogeneous novel magnetic SnFe_2_O_4_@SiO_2_–SO_3_H MNPs was checked for the Hantzsch synthesis of polyhydroquinolines, and the various reaction conditions were optimized in terms of the amount of catalyst, solvent, and temperature. A mixture of 4-chlorobenzaldehyde, dimedone, ethyl acetoacetate, and ammonium acetate was selected for optimization, as illustrated in Table 1. Table 1 shows that when the reaction was conducted without SnFe_2_O_4_@SiO_2_–SO_3_H, the reaction product yield percentage is traced until 240 min time (Table 1, entry 1). Initially, the influence of catalyst amount (0.005–0.012 g) on the reaction model was studied (Table 1, entries 4–7). Short reaction time and excellent yield were obtained in 0.012 g of the SnFe_2_O_4_@SiO_2_–SO_3_H in ethanol under reflux conditions (Table 1, entry 8). To illustrate the effect of the catalytic performance of SnFe_2_O_4_@SiO_2_–SO_3_H, the catalytic activity of its parents including SnFe_2_O_4_ and SnFe_2_O_4_@SiO_2_ were investigated, as illustrated in Table 1. It is obvious from Table 1 that SnFe_2_O_4_@SiO_2_–SO_3_H shows higher activity than its parent catalysts in Hantzsch reaction and when the SnFe_2_O_4_ and SnFe_2_O_4_@SiO_2_ were used as the catalyst, moderate yields were obtained. Then, the effects of various solvents were studied to test the model reaction (Table 1, entries 9–14). It is evident from entry 9 that the use of ethanol increases the yield of the product (25 min, 99%). Moreover, the other evaluation of solvents showed that reaction time is longer and the percentage of the product is lower. It is evident from entry 9 that the reaction at 80 °C is considered better than that at room temperature. Finally, the maximum performance and efficiency in terms of reaction time, percentage of products, solvent, and temperature were obtained in the model reaction using 0.012 g of SnFe_2_O_4_@SiO_2_–SO_3_H in ethanol under reflux conditions.

**Table 1:** Optimization of the reaction conditions for the Hantzsch condensation of 4-chlorobenzaldehyde and dimedone, ethyl acetoacetate, and ammonium acetate as the model reaction for the synthesis of polyhydroquinolines

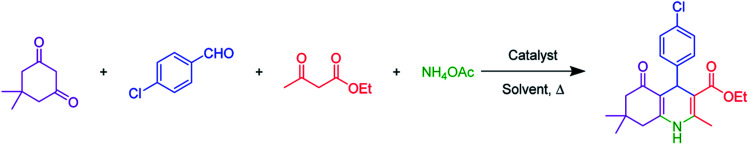						
Entry	Catalyst	Amount catalyst (mg)	Solvent	Temperature (°C)	Time (min)	Yield^a^^,^^b^ (%)
1	—	—	EtOH	Reflux	4 h	Trace
2	SnFe_2_O_4_	12	EtOH	Reflux	20	37
3	SnFe_2_O_4_@SiO_2_	12	EtOH	Reflux	20	33
4	SnFe_2_O_4_@SiO_2_–SO_3_H	5	EtOH	Reflux	20	29
5	SnFe_2_O_4_@SiO_2_–SO_3_H	7	EtOH	Reflux	20	67
6	SnFe_2_O_4_@SiO_2_–SO_3_H	9	EtOH	Reflux	20	87
7	SnFe_2_O_4_@SiO_2_–SO_3_H	10	EtOH	Reflux	20	91
8	SnFe_2_O_4_@SiO_2_–SO_3_H	12	EtOH	Reflux	20	99
9	SnFe_2_O_4_@SiO_2_–SO_3_H	12	EtOH	Reflux	20	99
10	SnFe_2_O_4_@SiO_2_–SO_3_H	12	EtOH	Reflux	20	87
11	SnFe_2_O_4_@SiO_2_–SO_3_H	12	H_2_O	Reflux	20	81
12	SnFe_2_O_4_@SiO_2_–SO_3_H	12	MeOH	Reflux	20	92
13	SnFe_2_O_4_@SiO_2_–SO_3_H	12	H_2_O:EtOH	Reflux	20	88
14	SnFe_2_O_4_@SiO_2_–SO_3_H	12	Solvent-free	80	20	90
15	SnFe_2_O_4_@SiO_2_–SO_3_H	12	EtOH	r.t.	20	Trace
16	SnFe_2_O_4_@SiO_2_–SO_3_H	12	EtOH	55	20	67
17	SnFe_2_O_4_@SiO_2_–SO_3_H	12	EtOH	70	20	88

a Isolated yield.

b Reaction conditions: 4-chlorobenzaldehyde (1 mmol), dimedone (1 mmol), ethyl acetoacetate (1 mmol), ammonium acetate (1.2 mmol), catalyst (mg) and solvent (3 mL).

After optimization, we examined various electron-withdrawing and electron-releasing benzaldehydes in the SnFe_2_O_4_@SiO_2_–SO_3_H-catalyzed Hantzsch reaction for the synthesis of polyhydroquinoline derivatives to identify the generality and the high proficiency of the catalytic system (Table 2). It is evident from Table 2 that a variety of polyhydroquinoline derivatives were synthesized with values of melting point, yield, and reaction time. As shown in table, both electron-withdrawing and electron-releasing benzaldehydes produced the corresponding derivatives with excellent yields and short reaction times, but, the reaction with electron-withdrawing benzaldehydes is considered faster than the one with electron-donating benzaldehydes.

**Table 2:** Hantzsch synthesis of polyhydroquinoline derivatives in the presence of SnFe_2_O_4_@SiO_2_–SO_3_H MNPs in ethanol under reflux conditions

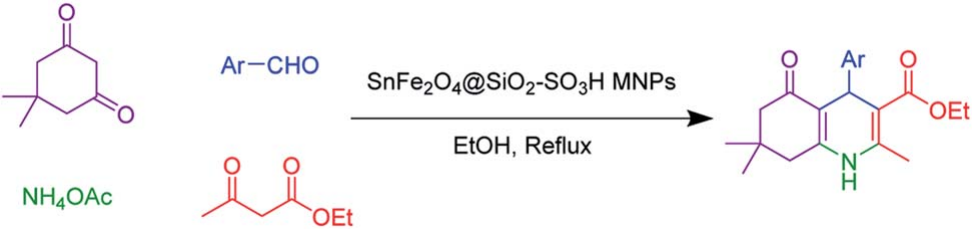						
Entry		Product	Time (min)	Yield^a^^,^^b^ (%)	Melting point	
					Measured	Literature
1	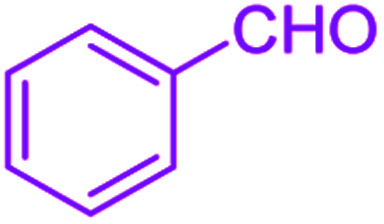	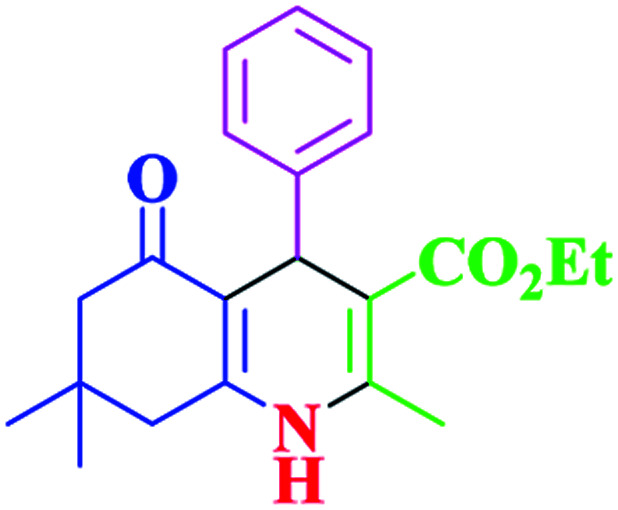	30	99	219–223	218–221 (ref. 38)
2	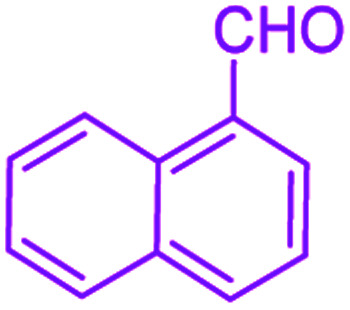	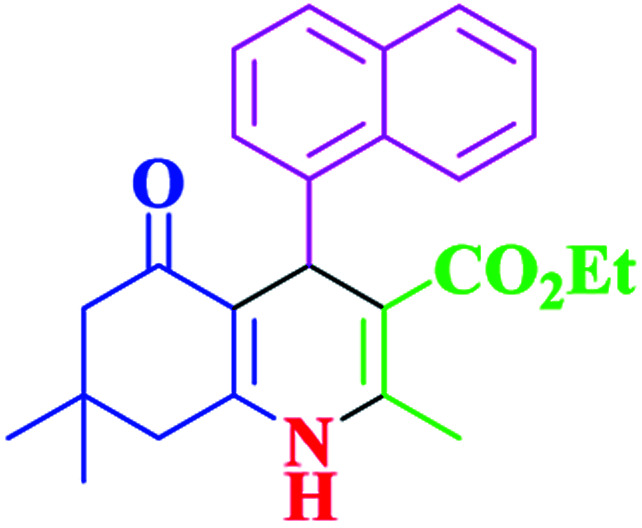	65	93	206–207	205–206 (ref. 38)
3	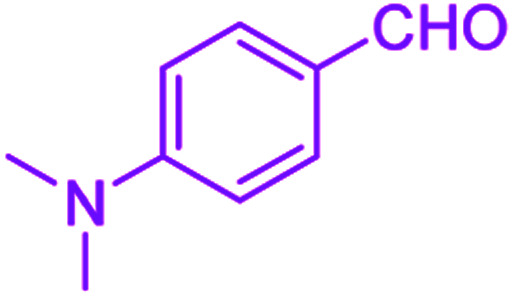	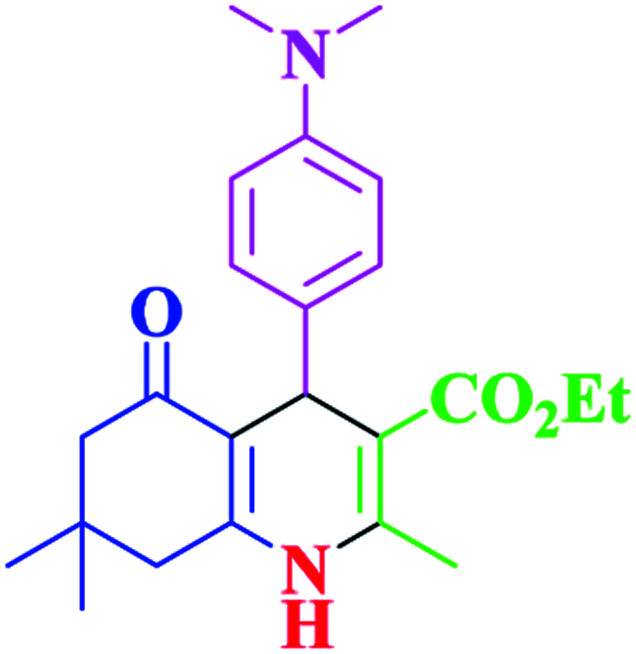	45	95	235–236	234–236 (ref. 38)
4	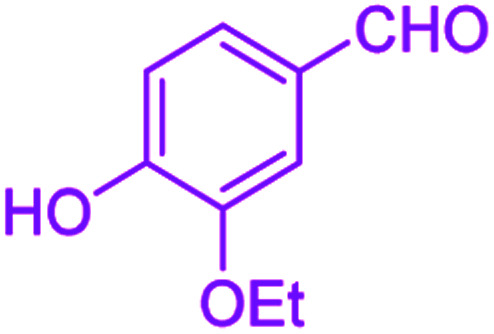	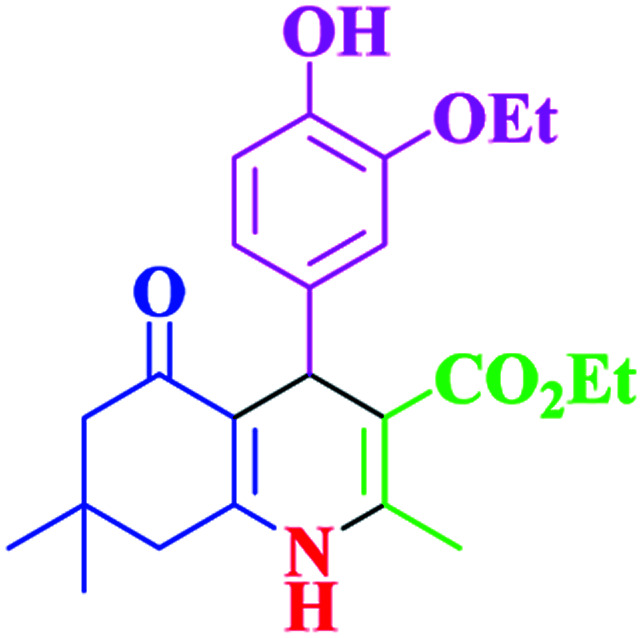	70	93	193–196	198–200 (ref. 39)
5	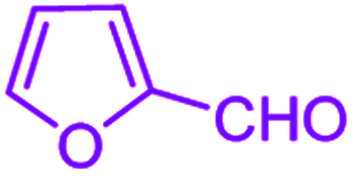	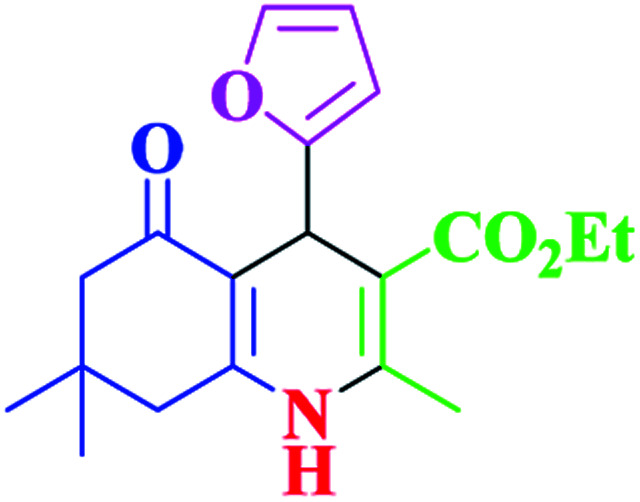	85	89	247–250	246–249 (ref. 38)
6	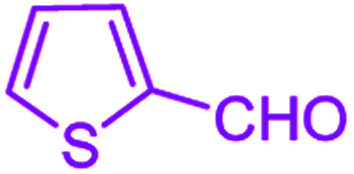	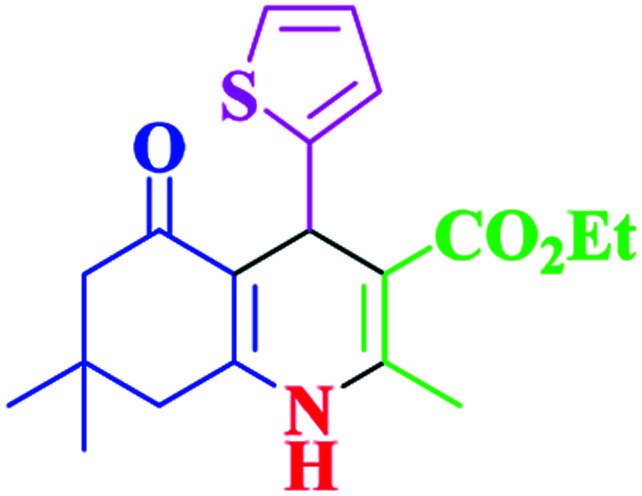	90	87	243–236	243–245 (ref. 38)
7	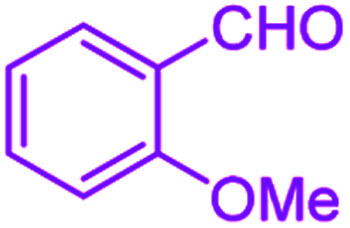	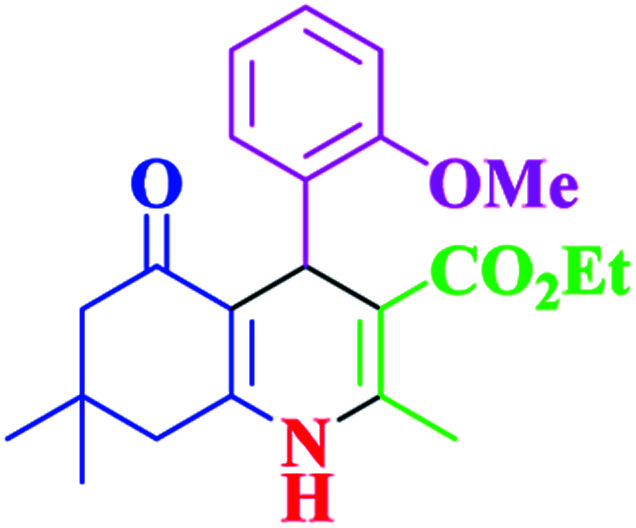	35	98	255–256	255–256 (ref. 38)
8	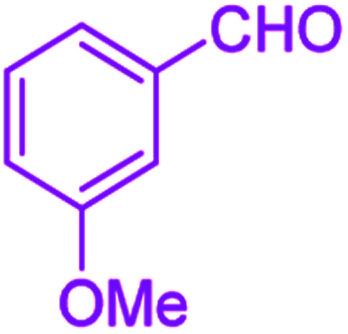	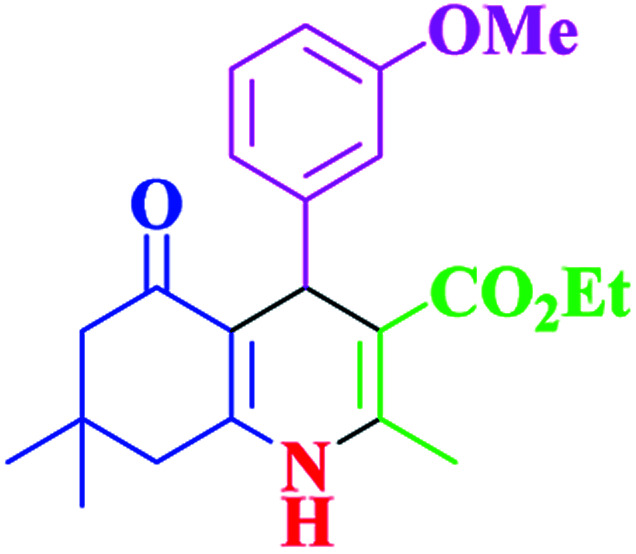	35	95	203–206	203–205 (ref. 38)
9	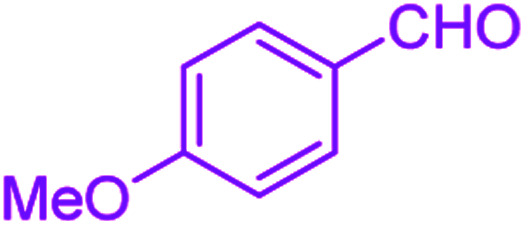	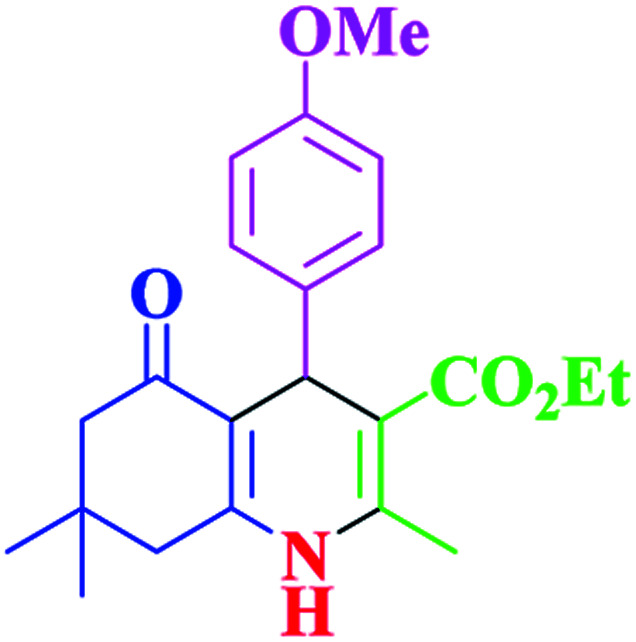	40	97	251–254	251–252 (ref. 40)
10	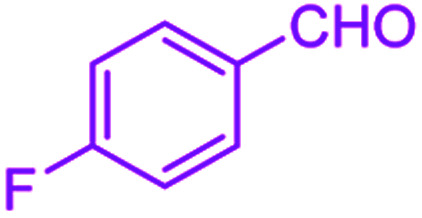	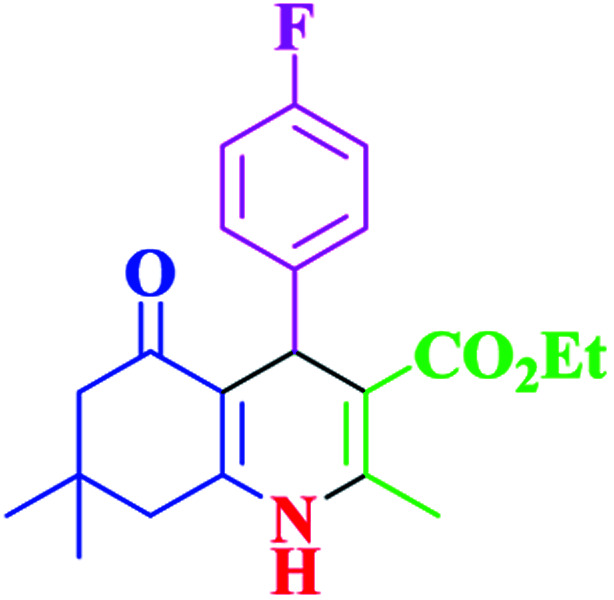	60	94	186–188	186–189 (ref. 38)
11	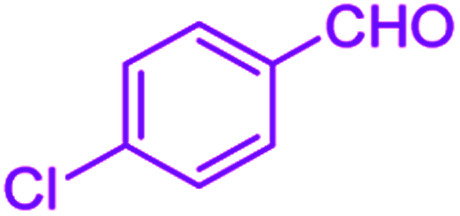	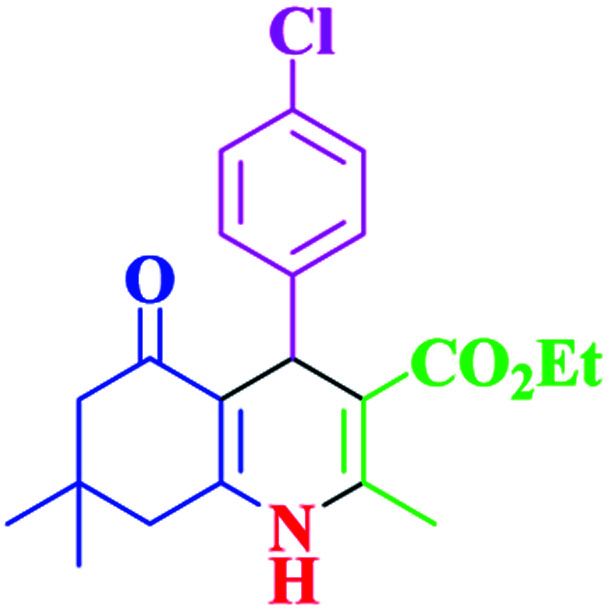	20	99	237–239	240–242 (ref. 38)
12	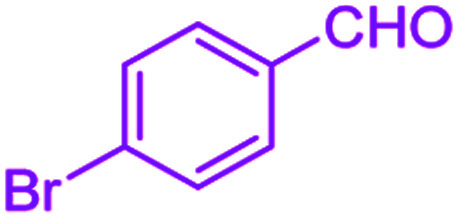	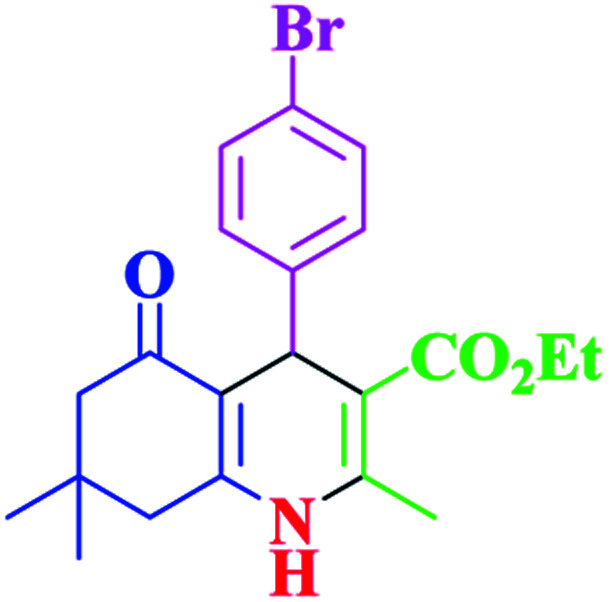	35	95	250–253	252–253 (ref. 40)
13	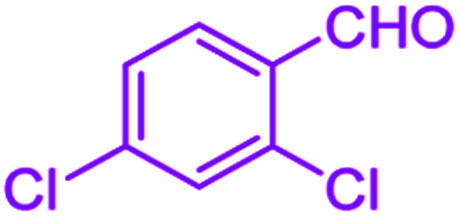	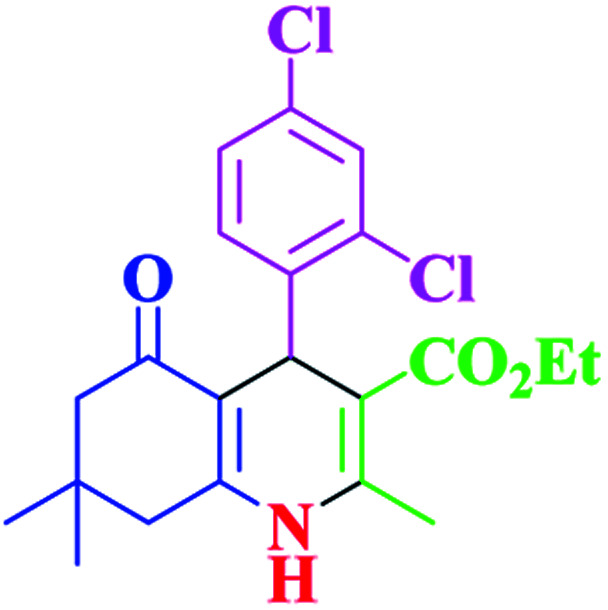	40	96	243–246	242–244 (ref. 38)
14	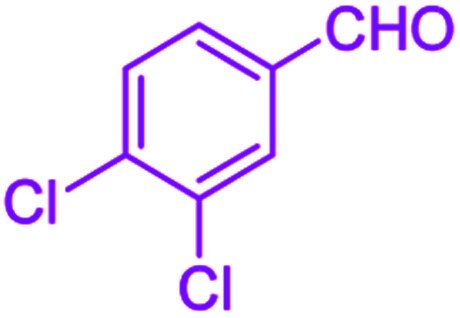	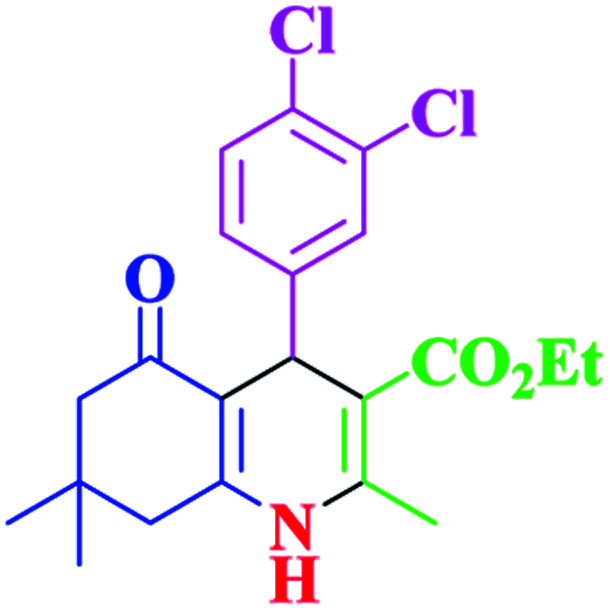	35	98	201–203	201–203 (ref. 41)

a Isolated yield.

b Reaction conditions: aromatic aldehyde (1 mmol), dimedone (1 mmol), ethyl acetoacetate (1 mmol), ammonium acetate (1.2 mmol), SnFe_2_O_4_@SiO_2_–SO_3_H MNPs (12 mg) in ethanol (3 mL) under reflux conditions.

### 3.3. Reaction mechanism

**Scheme 2:**
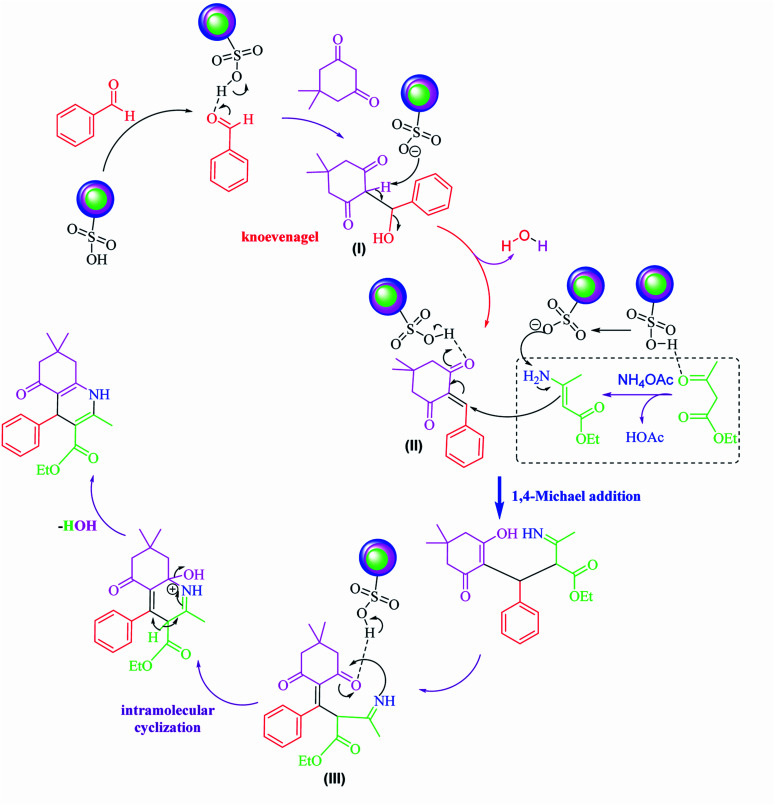
A possible mechanism for Hantzsch synthesis over SnFe_2_O_4_@SiO_2_–SO_3_H MNPs.

Based on our previous review on the synthesis of polyhydroquinolines,^1^ a possible transformation mechanism that accounts for the SnFe_2_O_4_@SiO_2_–SO_3_H MNPs as a novel heterogenized Brønsted–Lowry acid catalyst is illustrated in Scheme 2. It is proposed that the SnFe_2_O_4_@SiO_2_–SO_3_H catalyst due to its high Brønsted–Lowry acid property in the presence of the ethanol interacts with the oxygen present in the aldehyde functionality by the hydrogen bonding and leads to the activation of the carbonyl group. According to this mechanism, the nucleophilic addition by dimedone led to generating intermediate (I) which was followed by H_2_O molecule loss during Knoevenagel reaction and gave intermediate (II). Afterward, enamine (generated from the reaction of ethyl acetoacetate and ammonium acetate) and intermediate (II) underwent 1,4-Michael addition, affording the imine intermediate (III). Afterward, the imine intermediate underwent a cyclization reaction and, finally, afforded the targeted polyhydroquinolines products.

### 3.4. Catalyst reusability studies

**Fig. 9:**
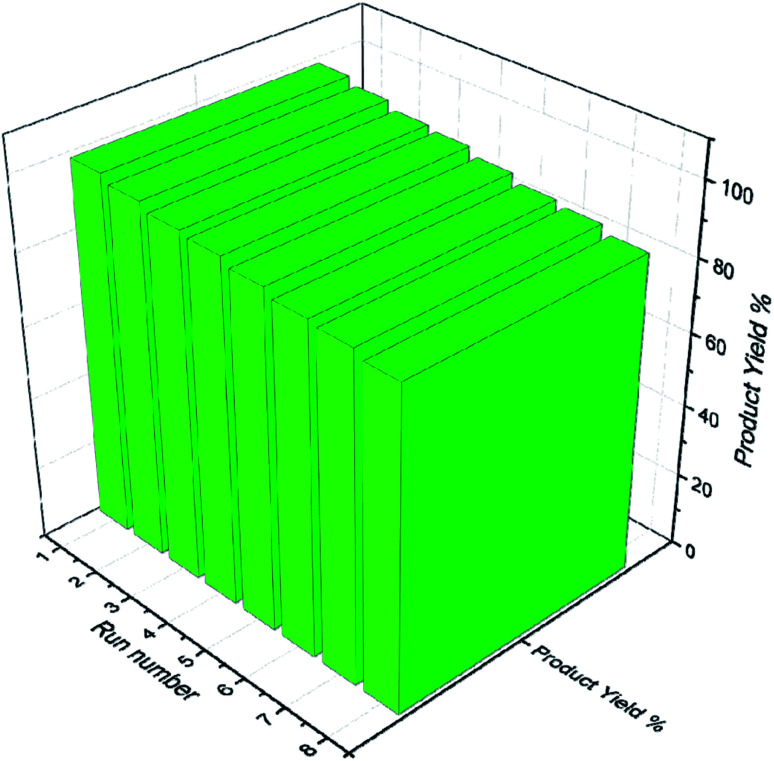
Reusability of SnFe_2_O_4_@SiO_2_–SO_3_H MNPs.

Catalysts having sulfides, metal oxides, and acids in their structure play an important and effective role in the industries like petroleum to fuel cleaning and other applications in precious materials. Hence, recycling the catalyst to prevent waste generation after use is one of the most important properties of catalysts. Nevertheless, recycling novel magnetic SnFe_2_O_4_@SiO_2_–SO_3_H was evaluated on the model reaction, and it was recycled up to 8 runs using an external magnet with a gradual decrease in activity from 99 to 87% in the corresponding product (Fig. 9).

**Fig. 10:**
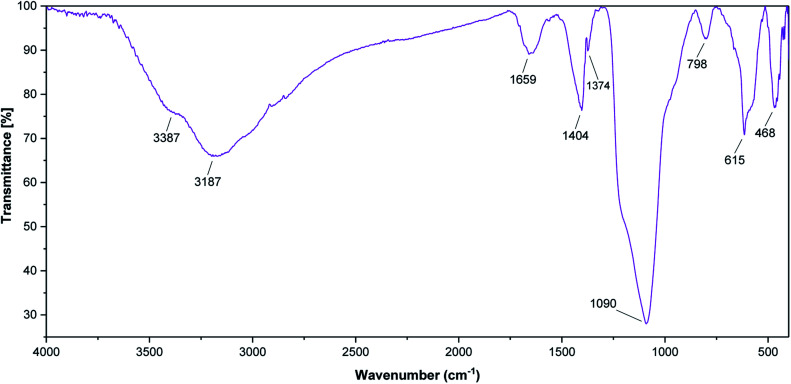
The FT-IR spectra of spent SnFe_2_O_4_@SiO_2_–SO_3_H MNPs.

**Fig. 11:**
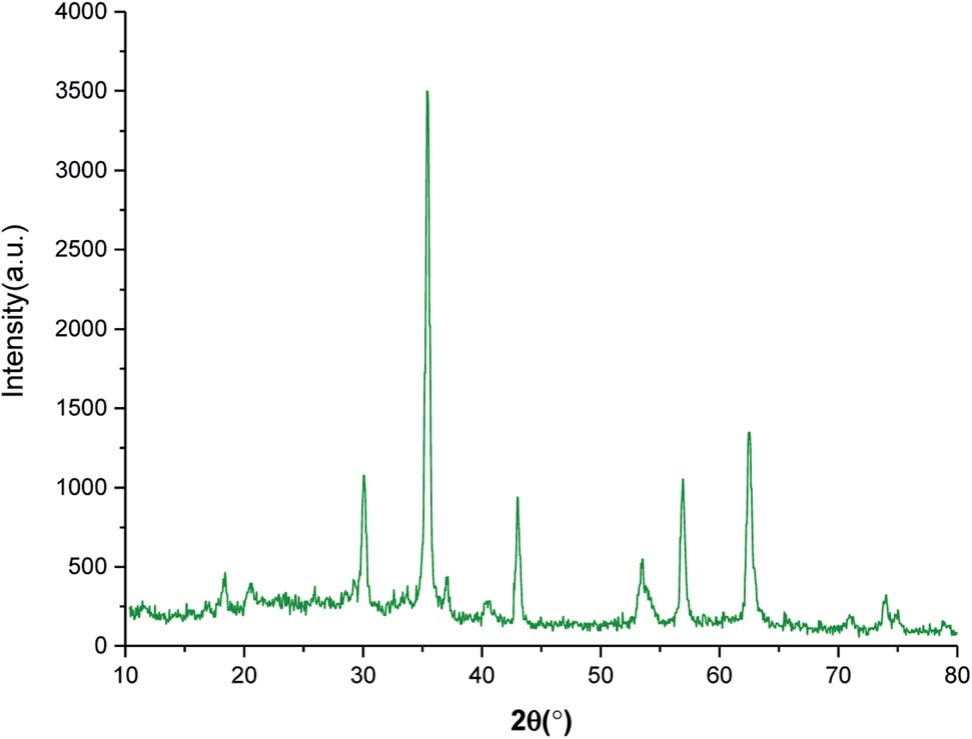
XRD pattern of spent SnFe_2_O_4_@SiO_2_–SO_3_H MNPs.

The stability of the recovered SnFe_2_O_4_@SiO_2_–SO_3_H MNPs was evaluated by FT-IR (Fig. 10) and P-XRD (Fig. 11) analysis. This investigation reveals that the obtained spectrums are in good agreement with the fresh catalyst results, and confirm that the composition and crystalline phase of the spent catalyst is not much affected even upon the 8th cycle. It supports the slight decrease in the yield of the catalytic products in the 8th cycle (87%) when compared to the fresh catalyst (99%). Our findings contribute to the development of new solid acid-based magnetic nanomaterials and the same strategy could be expanded to other industrial relevant metal catalyzed reactions.

## 4. Conclusion

In summary, we have successfully synthesized an effective and facile procedure for the synthesis of SnFe_2_O_4_@SiO_2_–SO_3_H as a magnetically recoverable nanocatalyst with characterization by a variety of techniques. The nanocatalyst indicated great performance in the Hantzsch reaction through a variety of aromatic aldehydes at loading as low as 0.012 g in ethanol under reflux conditions. The easy work-up procedure, usage of nontoxic solvent, excellent yield, short reaction time, good tolerance of our method toward various functional groups, and recycled and reused of catalyst by external magnet up to 8 runs with only a significant loss in the product yields are the several advantages for this method.

## Data availability

The data that support the findings of this study are available in the ESI.†

## Conflicts of interest

There are no conflicts to declare.

## Supplementary Material

RA-012-D2RA01202B-s001
