# Supplementary material for: Silica sulfuric acid coated on SnFe2O4 MNPs: synthesis, characterization and catalytic applications in the synthesis of polyhydroquinolines
Source: RSC Adv. 2022 May 12;12(23):14397–410. doi: 10.1039/d2ra01202b (PMC9097862; doi:10.1039/d2ra01202b)
Supplement: RA-012-D2RA01202B-s001 [file RA-012-D2RA01202B-s001.pdf]

Electronic Supplementary Material (ESI) for RSC Advances.
This journal is © The Royal Society of Chemistry 2022

Supporting Information

Silica Sulfuric Acid Coated on $SnFe_2O_4$ MNPs: Synthesis, Characterization and its Catalytic Applications in the Synthesis of Polyhydroquinolines

Soheila Esmaili [a], Ardeshir Khazaei [a,*], Arash Ghorbani-Choghamarani [a,*], Masoud Mohammadi[b]

[a]Department of Organic Chemistry, Bu–Ali Sina University, Hamedan, 6517838683, Iran.

[b]Department of Chemistry, Faculty of Science, Ilam University, P.O. Box 69315516, Ilam, Iran.

*correspondence to: Ardeshir Khazaei : ardeshir_khazaei@yahoo.com & Arash Ghorbani-Choghamarani, a.ghorbani@basu.ac.ir & arashghch58@yahoo.com

Spectral data:

Ethyl 2,7,7-trimethyl-5-oxo-4-phenyl-1,4,5,6,7,8-hexahydroquinoline-3-carboxylate:

FT-IR (KBr, cm^{-1}): 3288, 3218, 3083, 3027, 2961, 2930, 2908, 1698, 1644, 1610, 1484, 1381, 1309, 1071, 1030, 760 **S1**; ^{1}H NMR (300 MHz, DMSO-d_6): δ 0.86 (s, 3H), 1.03 (s, 3H), 1.14 (t, J= 3Hz, 3H), 1.97-2.22 (m, 2H), 2.34-2.53 (m, 2H), 3.99 (q, J= 3Hz, 2H), 4.89 (s, 1H), 7.08-7.20 (m, 5H), 9.1 (s, 1H) **S2**.

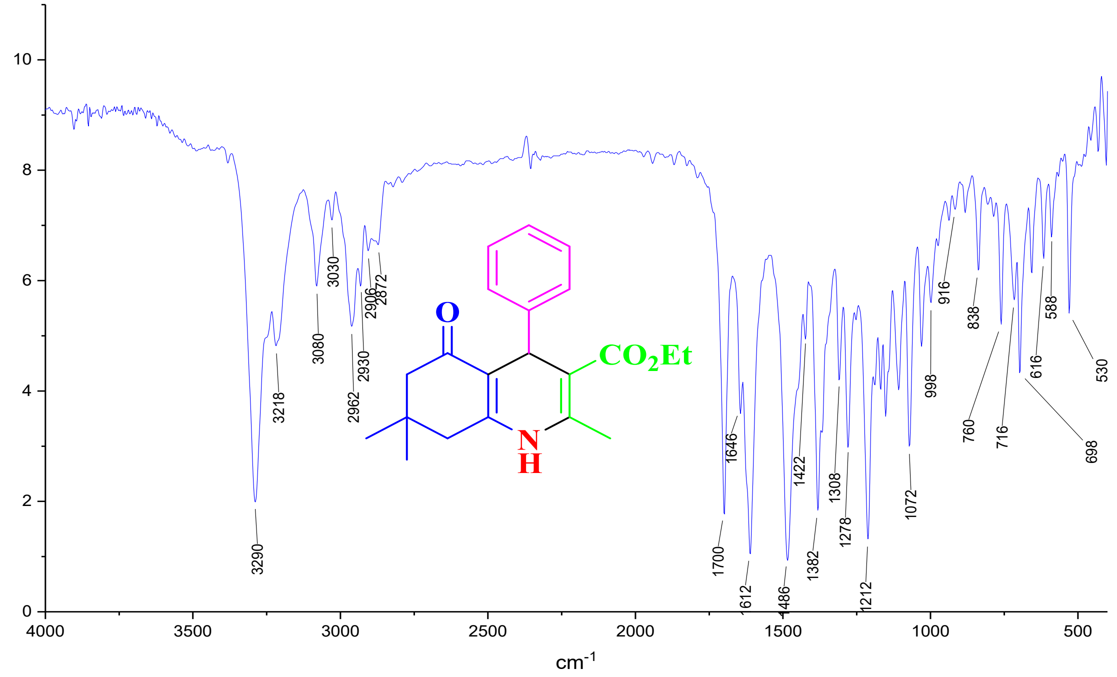

S1. FT-IR of Ethyl 2,7,7-trimethyl-5-oxo-4-phenyl-1,4,5,6,7,8-hexahydroquinoline-3-carboxylate carboxylate in KBr.

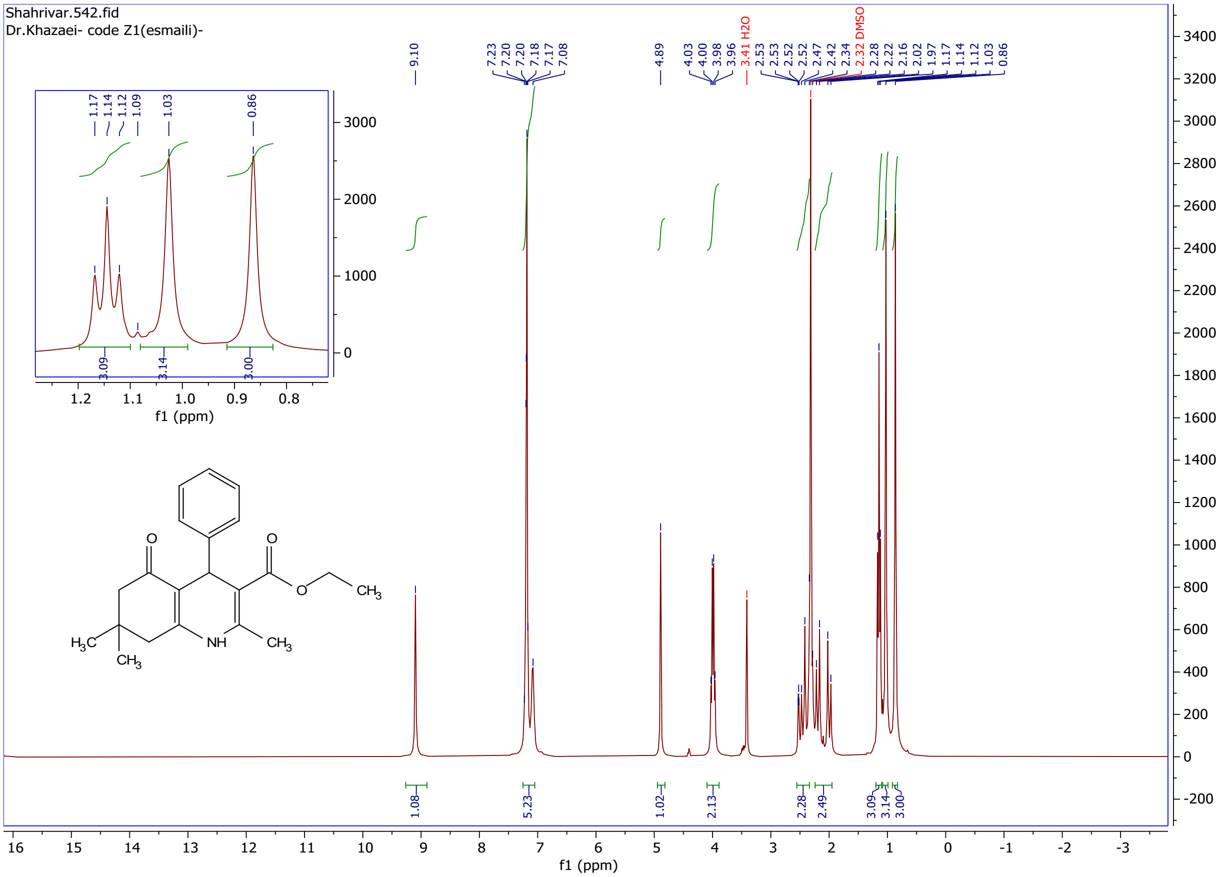

S2. ^{1}H NMR of Ethyl 2,7,7-trimethyl-5-oxo-4-phenyl-1,4,5,6,7,8-hexahydroquinoline-3-carboxylate carboxylate in DMSO.

Ethyl 4-(2,4-dichlorophenyl)-2,7,7-trimethyl-5-oxo-1,4,5,6,7,8-hexahydroquinoline-3-carboxylate

FT-IR (KBr, cm^{-1}): 3283, 3242, 3207, 3077, 2958, 28921705, 1648, 1608, 1493, 1380, 1280, 1213, 1152, 1107, 1073, 861 **S3** ; ^{1}H NMR (300 MHz, DMSO-d_6): δ 0.85 (s, 3H), 1.02 (s, 3H), 1.1 (t, J= 3Hz, 3H), 1.91-2.45 (m, 4H), 3.95 (m, 2H), 5.17 (s, 1H), 7.3 (s, 2H), 7.37 (s, 1H), 9.17 (s, 1H) **S4.**

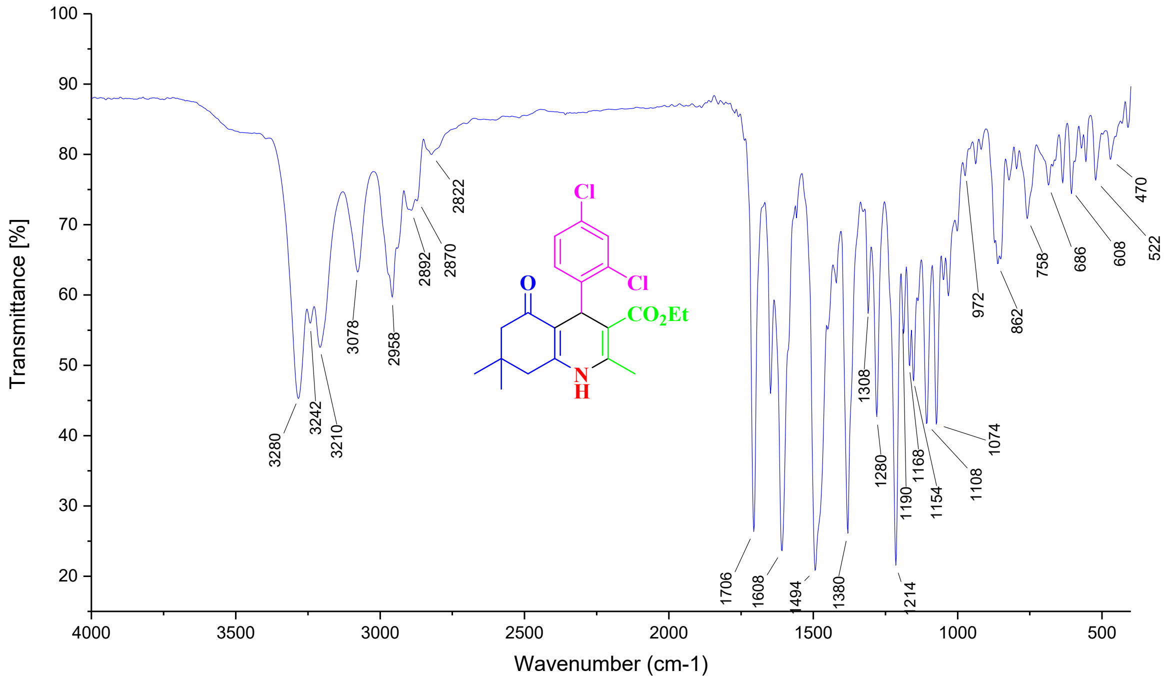

S3. FT-IR of Ethyl 4-(2,4-dichlorophenyl)-2,7,7-trimethyl-5-oxo-1,4,5,6,7,8-hexahydroquinoline-3-carboxylate in KBr.

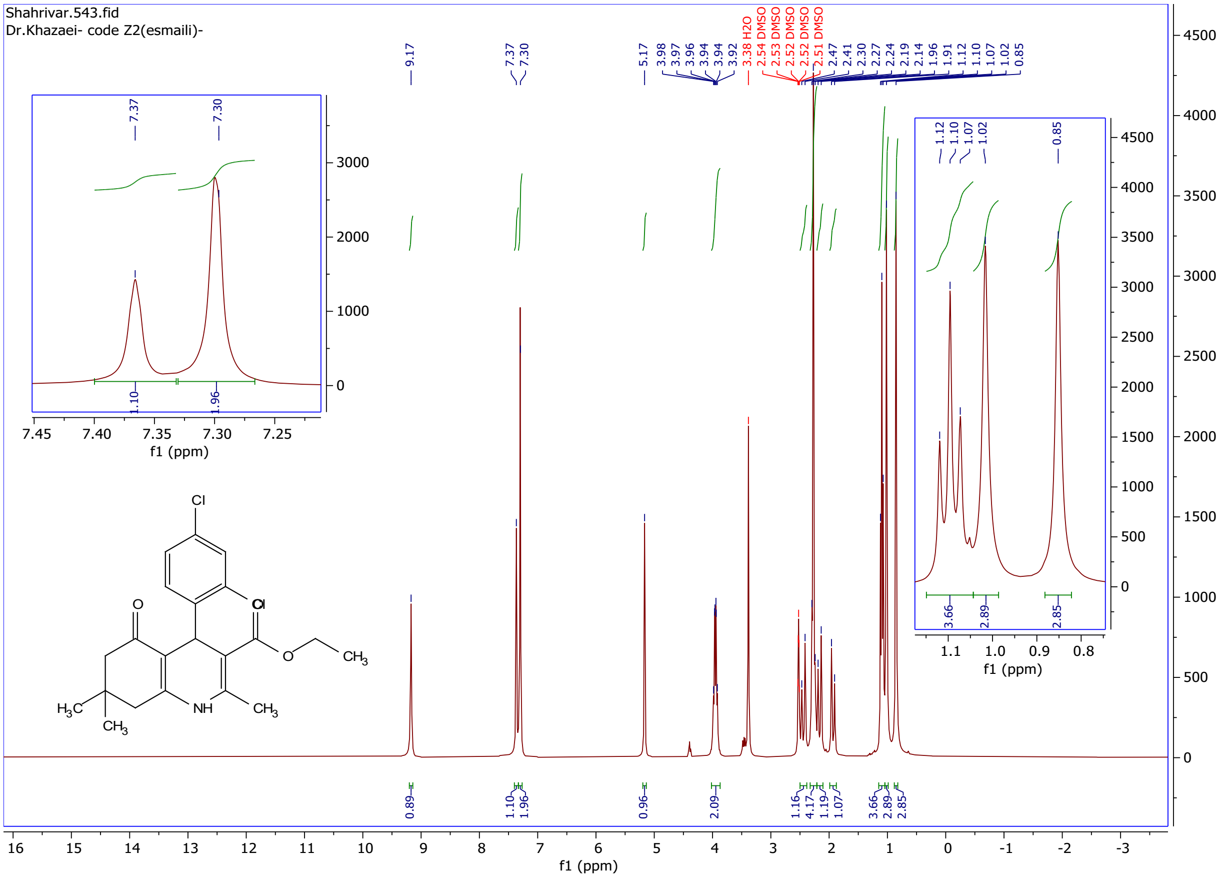

S4. ^{1}H NMR of Ethyl 4-(2,4-dichlorophenyl)-2,7,7-trimethyl-5-oxo-1,4,5,6,7,8-hexahydroquinoline-3-carboxylate in DMSO.

Ethyl 4-(3-ethoxy-4-hydroxyphenyl)-2,7,7-trimethyl-5-oxo-1,4,5,6,7,8-hexahydroquinoline-3-carboxylate

FT-IR (KBr, cm^{-1}): 3441, 3283, 3201, 3077, 2959, 2893, 1688, 1614, 1489, 1380, 1270, 1217, 1171, 1151, 1074, 1042, 784 **S5**; ^{1}H NMR (300 MHz, DMSO-d_6): δ 0.89 (s, 3H), 1.03 (s, 3H), 1.17 (t, J= 3Hz, 3H), 1.31 (t, J= 3Hz, 3H), 1.97-2.46 (m, 7H), 3.90-4.04 (m, 4H), 4.76 (s, 1H), 6.52 (d, *J= 9*, 1H), 6.6 (d, *J=9*, 1H), 6.71 (s, 1H), 8.57 (s, 1H), 9.1 (s, 1H) **S6.**

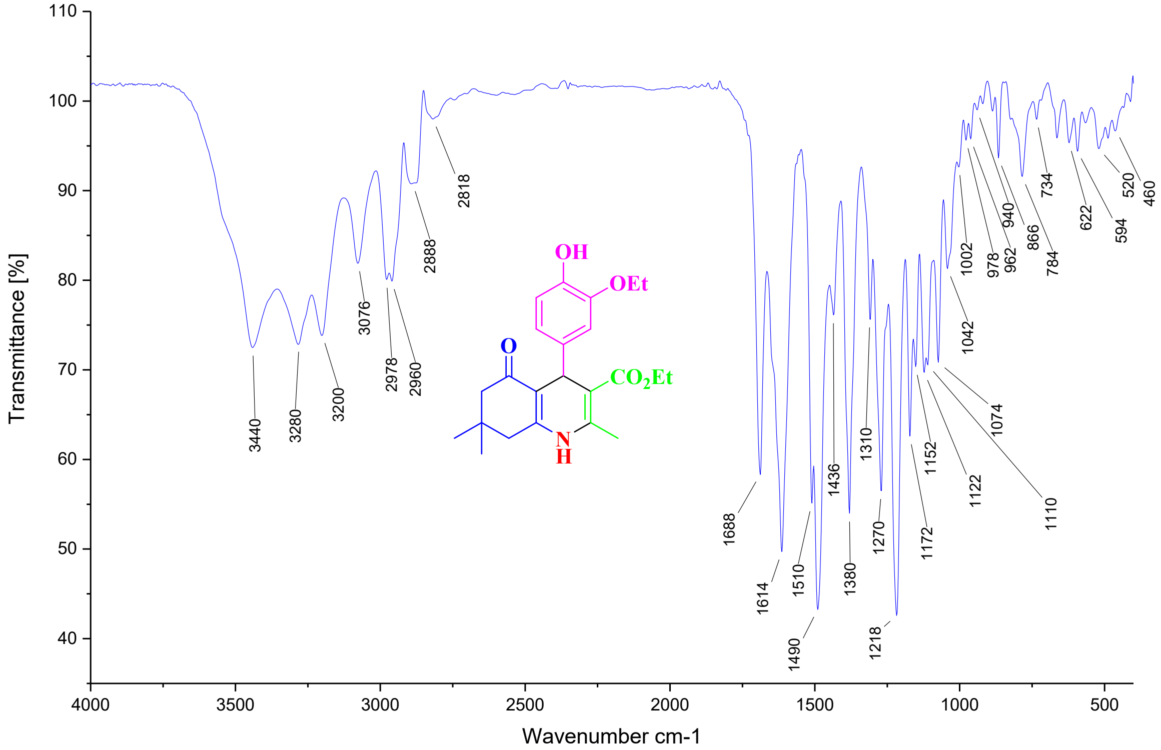

S5. FT-IR of Ethyl 4-(3-ethoxy-4-hydroxyphenyl)-2,7,7-trimethyl-5-oxo-1,4,5,6,7,8-hexahydroquinoline-3-carboxylate in KBr.

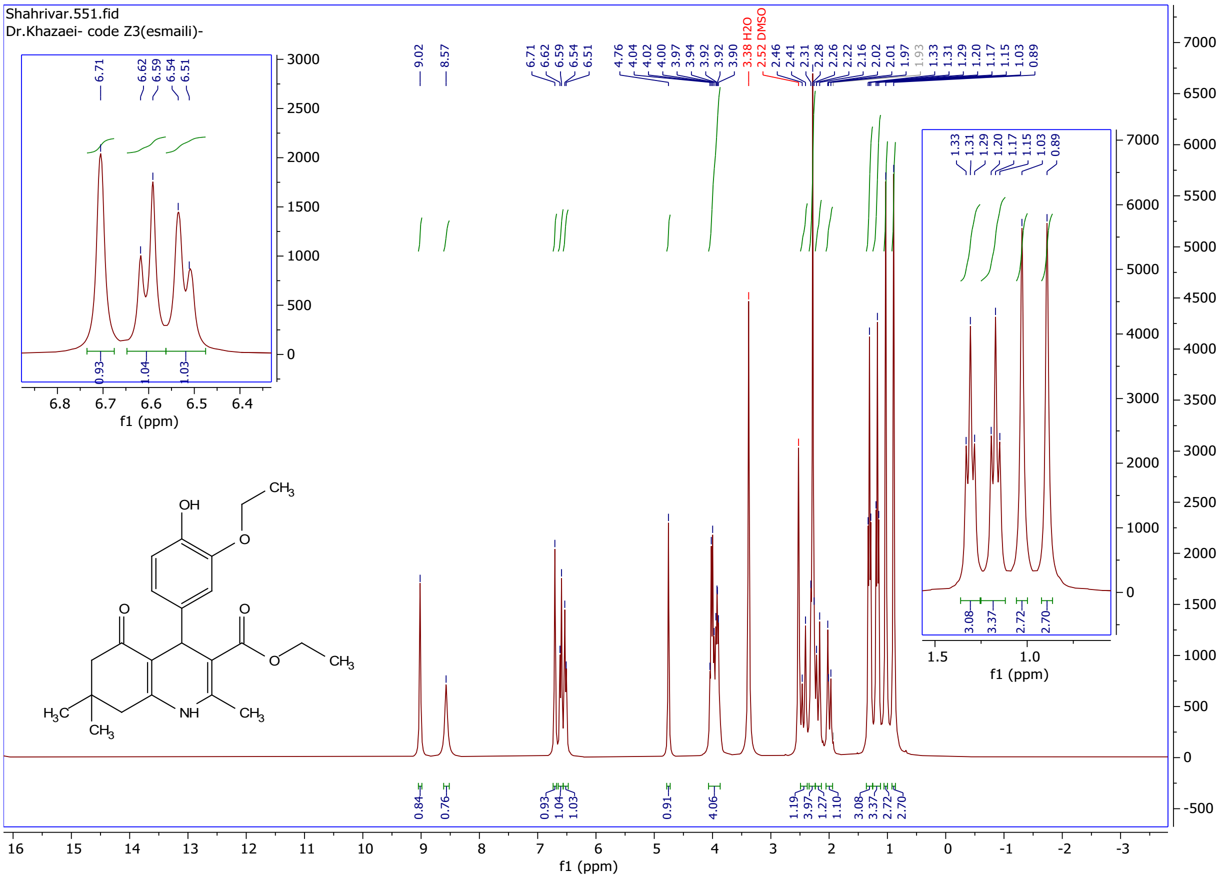

S6. ^{1}H NMR of Ethyl 4-(3-ethoxy-4-hydroxyphenyl)-2,7,7-trimethyl-5-oxo-1,4,5,6,7,8-hexahydroquinoline-3-carboxylate in DMSO.

Ethyl 2,7,7-trimethyl-5-oxo-4-(p-tolyl)-1,4,5,6,7,8-hexahydroquinoline-3-carboxylate

FT-IR (KBr, cm^{-1}): 3275, 3245, 3207, 3078, 2960, 2932, 2870, 1701, 1647, 1605, 1493, 1380, 1281, 1215, 1167, 1072, 1031, 530 **S7**; ^{1}H NMR (300 MHz, DMSO-d_6): δ 0.87 (s, 3H), 1.02 (s, 3H), 1.15 (t, 3H), 1.95-2.01 (d, J= 18 Hz, 2H), 2.15-2.32 (m, 8H), 2.41 -2.53 (m, 2H), 3.95-4.02 (q, 2H), 4.83 (s, 1H), 6.98 - 7.07 (m, *4*H), 9.05 (s, 1H) **S8**.

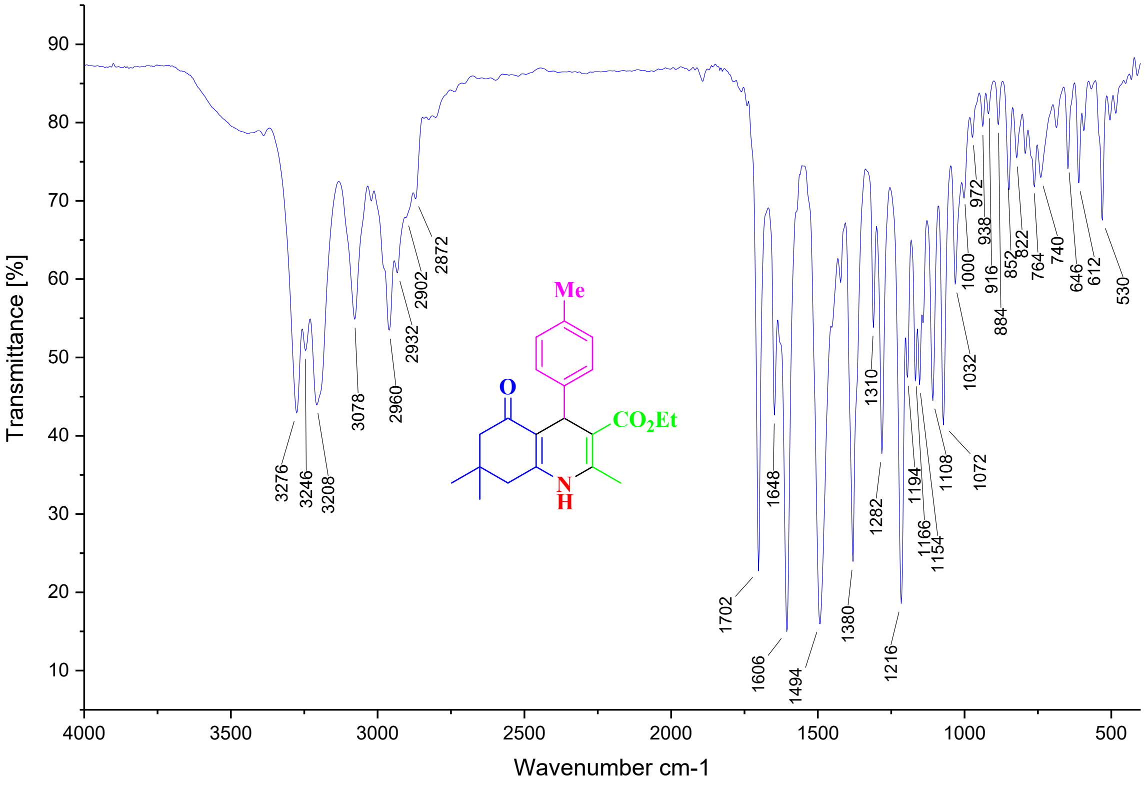

S7. FT-IR of Ethyl 2,7,7-trimethyl-5-oxo-4-(p-tolyl)-1,4,5,6,7,8-hexahydroquinoline-3-carboxylate in KBr.

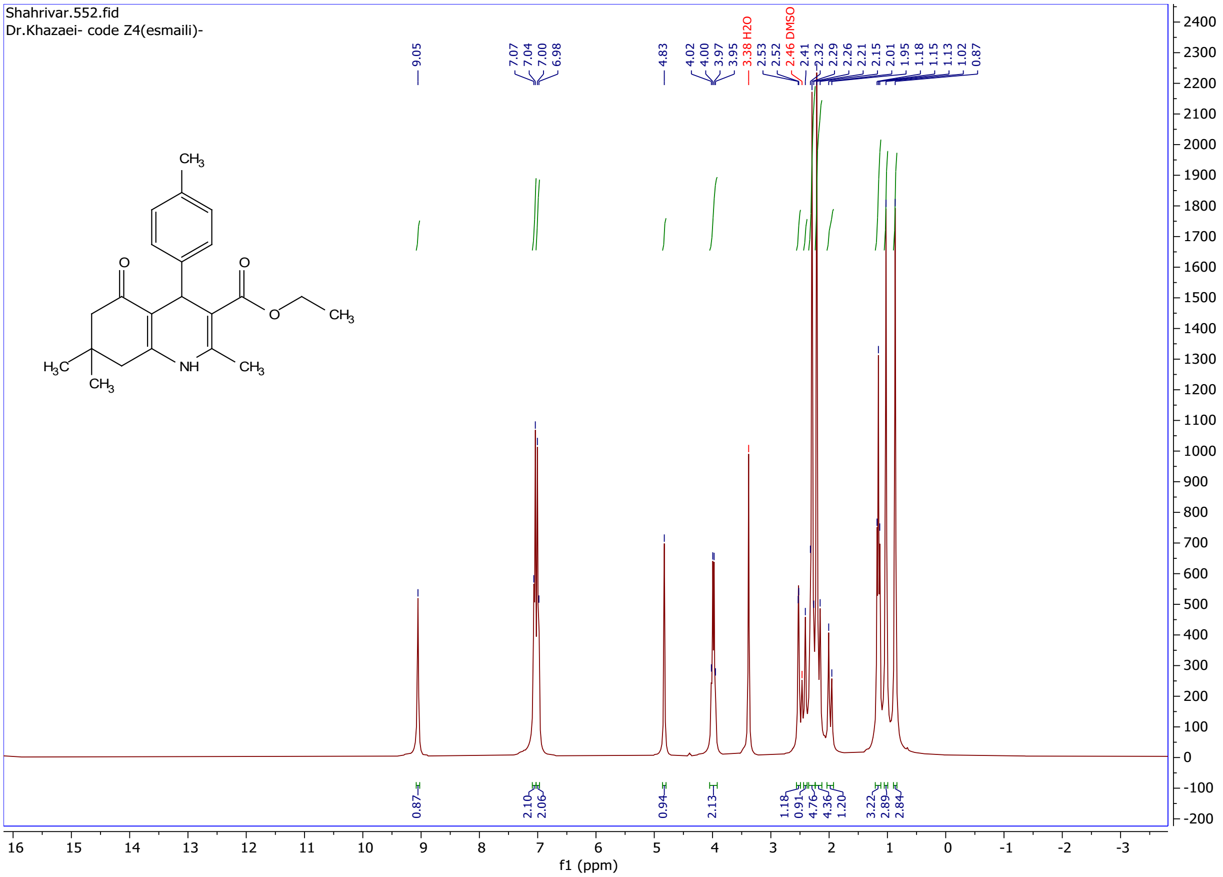

S8. ^{1}H NMR of Ethyl 2,7,7-trimethyl-5-oxo-4-(p-tolyl)-1,4,5,6,7,8-hexahydroquinoline-3-carboxylate in DMSO.

Ethyl 4-(4-(dimethylamino)phenyl)-2,7,7-trimethyl-5-oxo-1,4,5,6,7,8-hexahydroquinoline-3-carboxylate:

FT-IR (KBr, cm^{-1}): 3280, 3206, 3078, 2956, 2885, 2800, 1701, 1689, 1606, 1517, 1489, 1379, 1279, 1221, 1168, 1072 **S9** ; ^{1}H NMR (300 MHz, DMSO-d_6): δ 0.89 (s, 3H), 1.03 (s, 3H), 1.17 (t, J= 3Hz, 3H), 1.95-2.45 (m, 8H), 3.99 (q, J= 3Hz, 2H), 4.75 (s, 1H), 6.55-6.58 (d, *J*= *9*, 1H), 6.96-6.99 (d, *J*=*9*, 1H), 8.98 (s, 1H) **S10**.

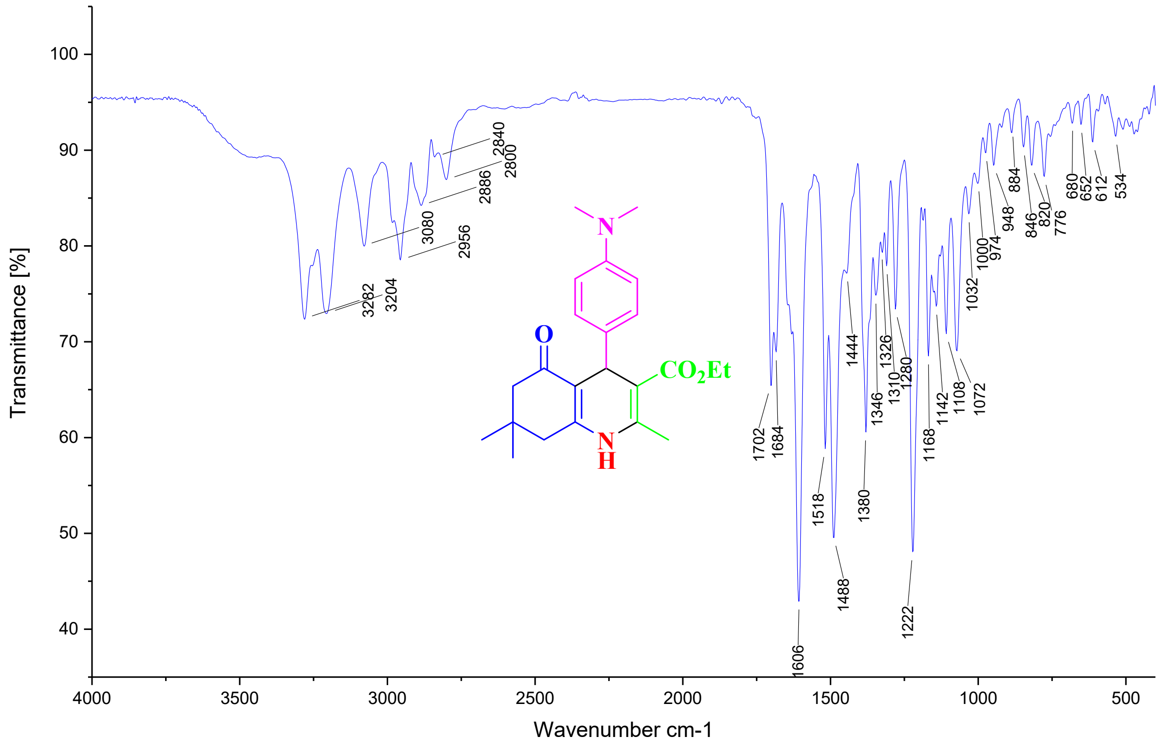

S9. FT-IR of Ethyl 4-(4-(dimethylamino)phenyl)-2,7,7-trimethyl-5-oxo-1,4,5,6,7,8-hexahydroquinoline-3-carboxylate in KBr.

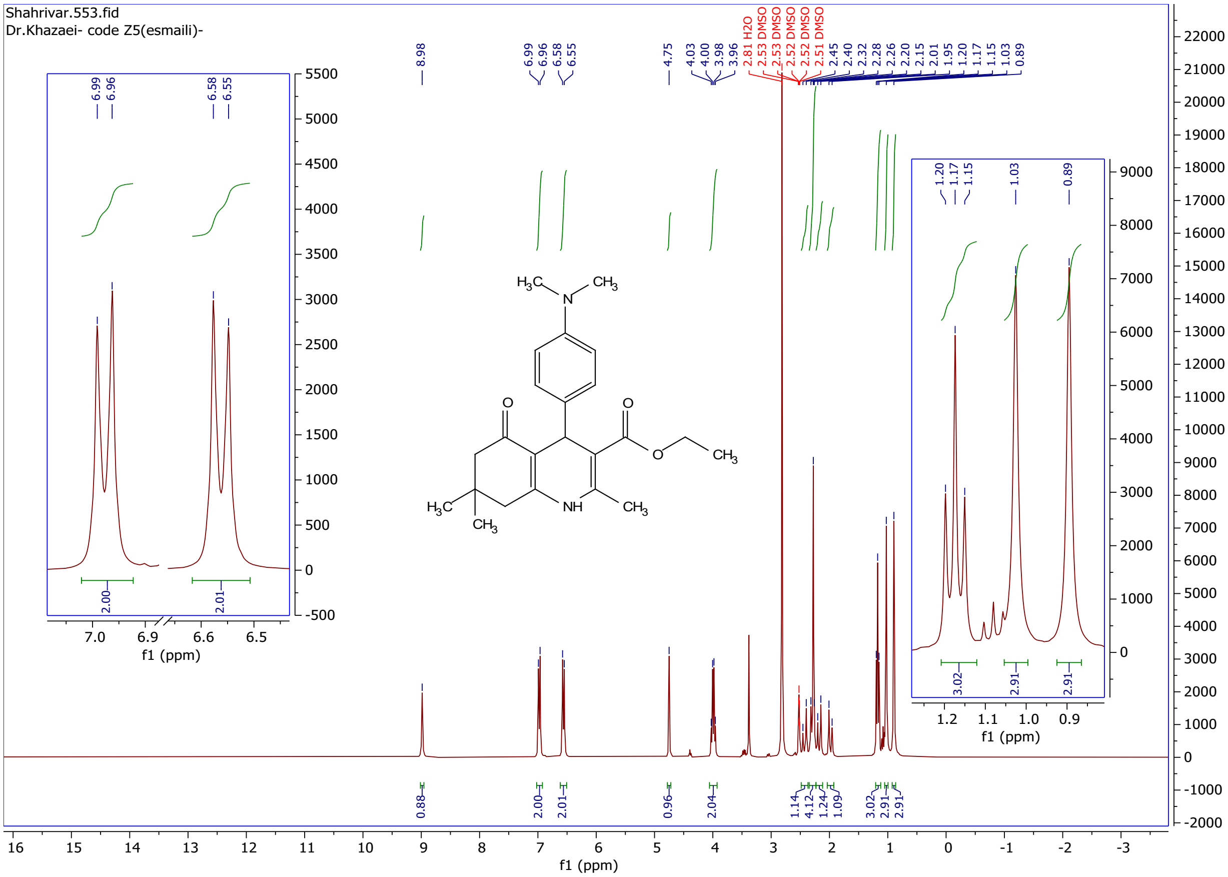

S10. ^{1}H NMR of Ethyl 4-(4-(dimethylamino)phenyl)-2,7,7-trimethyl-5-oxo-1,4,5,6,7,8-hexahydroquinoline-3-carboxylate in DMSO.

Ethyl 4-(2-methoxyphenyl)-2,7,7-trimethyl-5-oxo-1,4,5,6,7,8-hexahydroquinoline-3-carboxylate:

FT-IR (KBr, cm^{-1}): 3284, 3210, 3078, 2964, 2895, 1689, 1610, 1487, 1380, 1214, 1153, 1073, 1028, 751 **S11**; ^{1}H NMR (301 MHz, DMSO-d6): δ 0.86 (s, 3H), 1.02 (s, 3H), 1.13 (t, J= 3Hz, 3H), 3.21 (s, 3H), 1.88-2.45 (m, 8H), 3.71 (s, 3H), 3.9-3.96 (m, 2H), 5.06 (s, 1H), 6.75-6.87 (m, 2H), 7.04-7.13 (m, 2H), 8.98 (s, 1H) **S12**.

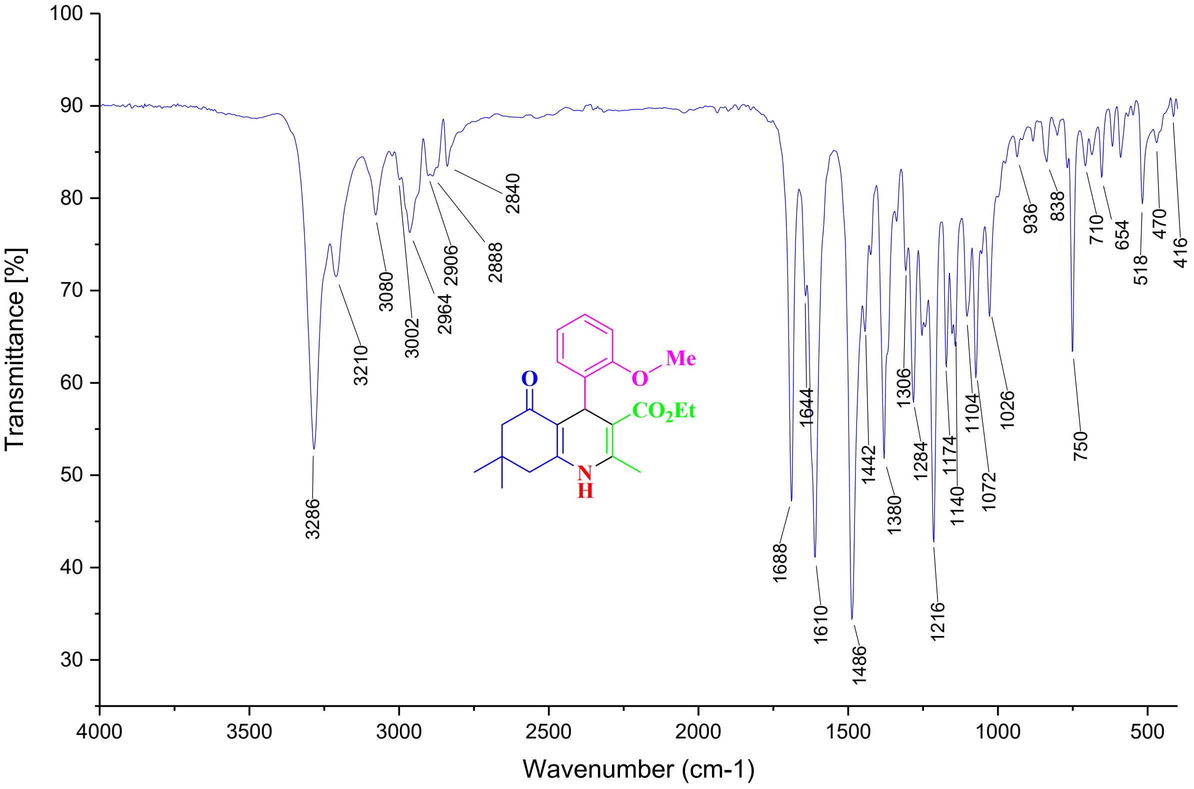

S11. FT-IR of Ethyl 4-(2-methoxyphenyl)-2,7,7-trimethyl-5-oxo-1,4,5,6,7,8-hexahydroquinoline-3-carboxylate in KBr.

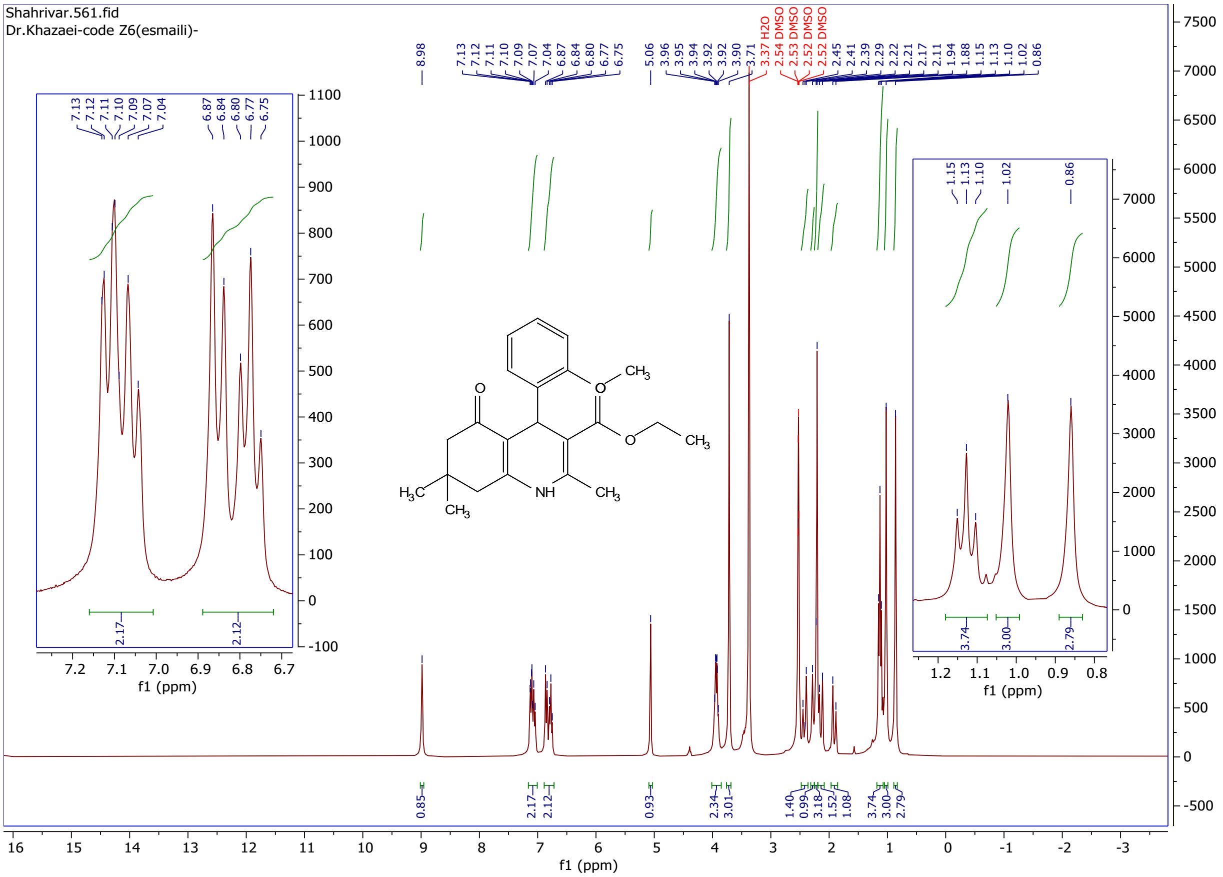

S12. ^{1}H NMR Ethyl 4-(2-methoxyphenyl)-2,7,7-trimethyl-5-oxo-1,4,5,6,7,8-hexahydroquinoline-3-carboxylate in DMSO.
